# Potential molecular pathways and therapeutic implications of rapid-acting antidepressants on myelin biology: a scoping review

**DOI:** 10.3389/fnins.2025.1690318

**Published:** 2026-01-12

**Authors:** Antonio Inserra, Colin J. Murray, Antonella Campanale, Jared VanderZwaag, Marie-Ève Tremblay

**Affiliations:** 1 Department of Neuroscience, Imaging and Clinical Sciences, G. d'Annunzio University of Chieti-Pescara, Chieti, Italy; 2 Behavioral Neuroscience Laboratory, Postgraduate Program in Health Sciences, University of South Santa Catarina (UNISUL), Tubarão, Brazil; 3 School of Medical Sciences, Faculty of Health, University of Victoria, Victoria, BC, Canada; 4 Institute on Aging and Lifelong Health (IALH) and Center for Advanced Materials and Related Technologies (CAMTEC), University of Victoria, Victoria, BC, Canada; 5 Department of Psychiatry, McGill University, Montreal, QC, Canada

**Keywords:** rapid-acting antidepressants, myelination, neuroplasticity, oligodendrocytes, neuronal activity-dependent myelination, ketamine

## Abstract

**Background:**

Emerging evidence indicates that rapid-acting antidepressants (RAADs)—including ketamine and serotonergic psychedelics- may affect myelin homeostasis, aside from producing fast-onset, sustained improvements in several psychiatric disorders.

**Methods:**

A systematic search of PubMed (MEDLINE), Web of Science, Europe PMC, Directory of Open Access Journals (DOAJ), and Google Scholar was conducted up to October 2025 for studies examining the effects of RAADs on myelination and oligodendrocyte biology, as well as associated molecular and cellular mechanisms.

**Main body:**

Forty-one studies met the inclusion criteria: 12 in humans, 21 in animals, 7 *in vitro*, and one computational/theoretical. Thirty studies investigated ketamine and 11 serotonergic RAADs. Across models, RAADs modulate myelination in a dose- and exposure-dependent manner: therapeutic doses generally promote myelin integrity and oligodendrocyte maturation, while high or repeated doses, or neonatal exposure, can disrupt myelin structure and function, impair oligodendrocyte viability, and produce cognitive, affective, and neurotoxic side effects.

**Conclusion:**

Myelin regulation may represent a component of RAAD action, indicating that these agents could influence myelin biology. Further research is required to clarify the mechanisms underlying these effects, their potential implications for therapies aimed at preserving or restoring myelin integrity, and potential side effects. Their dose-dependent effects highlight the need for careful consideration of dosing and treatment duration.

## 1 Introduction

Myelin is a modified and extended lipid-rich plasma membrane of oligodendrocytes (OLs) in the central nervous system and Schwann cells in the peripheral nervous system of vertebrates, wrapping axons to provide electrical insulation and metabolic support (Ben Geren, 1954; Sherman and Brophy, 2005; Salzer et al., 2008). Myelin homeostasis exists in a multilayered equilibrium intimately linked to neuronal homeostasis, which is an essential prerequisite for health (de Faria et al., 2021).

Dysregulation of myelin homeostasis can occur (a) in response to stress exposure (Lutz et al., 2017; Yang et al., 2017), (b) in neurodevelopmental and psychiatric disorders (Hakak et al., 2001; Tkachev et al., 2003; Aston et al., 2005; Georgieva et al., 2006; Stewart et al., 2007), (c) in myelin-degenerative diseases (i.e., multiple sclerosis (MS), leukodystrophies, and peripheral neuropathies) (Stadelmann et al., 2019), (d) following brain injury, (e) during aging (Safaiyan et al., 2016), and (f) in neurodegenerative diseases like Parkinson’s and Alzheimer’s diseases (Papuć and Rejdak, 2018; Yang et al., 2023). The resulting demyelinated axons experience significant physiological alterations and molecular rearrangements, leading to impaired axo-OL signaling and axonal function, including altered conduction and neuronal excitability, degeneration, and ultimately, a progressive loss of cognitive and sensorimotor functions (Kujala et al., 1997; Gootjes et al., 2004). Currently available therapeutic strategies to prevent or reverse myelin degeneration associated with aging and neurodegenerative diseases remain limited. Therefore, more effective treatments are needed.

Among various pharmacological approaches under investigation, rapid-acting antidepressants (RAADs), which include serotonin (5-HT)2A receptor agonists such as psilocybin, lysergic acid diethylamide (LSD), and N, N-Dimethyltryptamine (DMT) (Inserra et al., 2021a), and the N-Methyl-D-Aspartate (NMDA) receptor antagonist ketamine (Krystal et al., 2023) have shown the ability to affect axonal myelination, OL function, the expression of myelin-related proteins and the gut-brain axis. Therefore, RAADs might represent novel candidate therapeutics to preserve, or restore, myelin homeostasis.

In this scoping review, we examine the available literature concerning the effects of RAADs on myelination and myelin homeostasis. We identify mechanistic insights, highlight existing knowledge gaps, and propose directions for future research to better understand the potential role of RAADs in modulating myelin-related processes relevant to psychiatric and neurodegenerative disorders.

## 2 Methods

### 2.1 Search strategy

We performed a systematic search of PubMed (MEDLINE), Web of Science, Europe PMC, Directory of Open Access Journals—DOAJ, and Google Scholar for studies published up to October 2025. The following search string was used (adapted to each database’s syntax):

(“myelin” OR “myelination” OR “oligodendrocyte” OR “oligodendrocyte precursor cell” OR “OPC” OR “oligodendrogenesis” OR “white matter” OR “demyelination” OR “remyelination”)

AND

(“rapid-acting antidepressant” OR “RAAD” OR “serotonergic psychedelic” OR “ketamine” OR “S-ketamine” OR “R-ketamine” OR “NMDA antagonist” OR “psilocybin” OR “LSD” OR “lysergic acid diethylamide” OR “DMT” OR “N, N-dimethyltryptamine” OR “ayahuasca”)

AND

(“mechanism” OR “molecular pathway” OR “cellular mechanism” OR “signaling” OR “effects”).

We limited results to peer-reviewed articles written in English, including original human, animal, and *in vitro* studies, provided they investigated the effects of RAADs on myelination-related outcomes or pathways involving OLs, OPCs, white matter (WM), or myelin plasticity. The reference lists of included studies and literature reviews (which are not included in this review) were manually searched to identify additional sources.

## 3 Results

### 3.1 Overview of included studies

A total of 41 studies were identified, including 12 human, 21 animal, 7 *in vitro*, and one theoretical modeling study. Among these, 30 focused on ketamine (Table 1) and 11 on the serotonergic RAADs LSD (8), 2,5-Dimethoxy-4-iodoamphetamine (DOI) (2), and psilocybin (1). One *in vitro* study investigated more than one serotonergic RAAD (LSD, 5-Cl-DMT, bufotenine, DMT, 5-MeO-DMT, 5-methoxytryptamine, mescaline, dimethyl DOM) (Table 2).

**Table 1:** Summary of experimental and clinical studies investigating the effects of ketamine and serotonergic psychedelics on white matter (WM) structure, myelination, and oligodendrocyte (OL) biology.

Model and characteristics Intervention Reference	Experimental methods Parameters evaluated	Results	Significance
Ketamine			
Studies with therapeutic regimens			
Male and female adult individuals with treatment-resistant depression (TRD) Ketamine (0.5 mg/kg, i.v.) (Vasavada et al., 2016)	Diffusion Magnetic Resonance Imaging (MRI)	Ketamine responders have greater post-infusion fractional anisotropy (FA) in the cingulum and forceps minor, and smaller radial diffusivity (RD) in the forceps compared to non-responders Improvements in depression scores 24 h post-ketamine infusion are associated with increased FA in the cingulum, decreased mean diffusivity (MD) and RD in the forceps minor, and decreased RD in the striatum	Ketamine’s antidepressant effects may involve enhanced myelination and white matter (WM) plasticity
Male and female individuals with TRD pre- and 4 h post ketamine infusion Ketamine (0.5 mg/kg, i.v.) (Sydnor et al., 2020)	Diffusion MRI	FA increases in the following WM bundles of the brain 4 h post-ketamine infusion: right cingulum bundle-hippocampal portion; right inferior longitudinal fasciculus; right uncinate fasciculus; left inferior longitudinal fasciculus; right inferior fronto-occipital fasciculus; left superior longitudinal fasciculus; left cingulum bundle-cingulate gyrus portion; right superior longitudinal fasciculus; left cingulum bundle-hippocampal portion; left inferior fronto-occipital fasciculus; corpus callosum-forceps minor; corpus callosum-forceps major; right cingulum bundle-cingulate gyrus portion; fornix Higher pre-infusion FA in the left cingulum bundle and left superior longitudinal fasciculus is associated with greater depression symptom improvement 24 h post-infusion Greater post-ketamine increases in WM FA are associated with poorer 24 h symptom reduction	The rapid WM diffusivity changes elicited by ketamine might have functional significance given their correlation with symptom improvements
Male and female adult individuals with TRD Ketamine (0.5 mg/kg, i.v.) (Langhein et al., 2022)	Diffusion MRI	Responders to ketamine have greater baseline tissue-specific FA in the left cingulum bundle compared to non-responders	Tissue-specific FA in the left cingulum bundle—may be a predictor of clinical response to ketamine Pre-existing myelin integrity and baseline WM microstructure might influence therapeutic efficacy in TRD
Male and female individuals with TRD and healthy controls 4 ketamine administrations (0.5 mg/kg, i.v.) (Taraku et al., 2023)	Diffusion MRI	Repeated ketamine in individuals with TRD leads to significant decreased neurite density index (NDI) in major WM tracts within the left occipital and left temporal lobes, including the posterior thalamic radiation, inferior longitudinal fasciculus, forceps major, and the retrolenticular part of the internal capsule Greater decreases in NDI in WM tracts passing through the internal capsule and superior longitudinal fasciculus significantly correlated with greater improvements in anhedonia	Repeated ketamine administration reduces neurite density in specific WM tracts, with these microstructural changes correlating with improvements in anhedonia in individuals with TRD
Male and female individuals with neuropathic pain Ketamine (titrated individually between 0.5 to 2 mg/kg/h, i.v., for 6 h) over 5 consecutive days followed by 6 weeks of oral ketamine (0.5 mg/kg) 3 times per day (Mills et al., 2024)	Diffusion MRI	Baseline fiber density of several large-scale frontal white matter pathways in the anterior limb of the internal capsule was positively correlated with greater pain relief following ketamine, including the fronto-pontine tract, thalamo-prefrontal tract, and anterior thalamic radiations. The baseline FD within the left mPFC-PCu/PCC (medial Prefrontal Cortex – Precuneus/Posterior Cingulate Cortex), and within the mPFC-PAG (periaqueductal gray) are inversely correlated with symptom improvement	Individual differences in WM microstructure may influence ketamine’s analgesic efficacy in neuropathic pain
Male and female individuals with MDD S-ketamine (0.25 mg/kg, i.v.) plus sertraline (escalating doses, 50, 100, and 150 mg/day) for 2 weeks (Liu et al., 2025)	Diffusion MRI	Repeated S-ketamine combined with escalating doses of sertraline does not reverse the widespread disrupted microarchitecture (TBSS, FA, MD) of WM present in individuals with MDD	WM abnormalities in MDD may be resistant to short-term combined intervention of S-ketamine plus sertraline
Infants with ventricular septal defects undergoing cardiopulmonary bypass for repair of ventricular septal defects Ketamine (2 mg/kg, i.p.) (Bhutta et al., 2012)	MRI with spectroscopy (MRS)	Ketamine administration leads to a significant decrease in choline and glutamate plus glutamine/creatine in frontal WM	Ketamine exposure during early development may alter frontal WM metabolism, potentially affecting membrane turnover or myelination processes
9–11-week-old male Sprague–Dawley rats Ketamine (25 mg/kg, i.p.) (Pascual-Antón et al., 2021)	Diffusion MRI Myelin basic protein (MBP) and Neurofilament 200 kDa, (NF200) immunohistochemistry with confocal microscopy Mbp and NF200 immunoreactivity	Ketamine increases MBP expression in the infralimbic cortex deep layers, and decreases RD across multiple brain regions: dorsal raphe nucleus-DRN-, ventral hippocampus, amygdala, thalamus, corpus callosum	Ketamine might enhance myelination in key WM tracts, which might be linked to the brain plasticity and antidepressant effects elicited
Adult male C57BL6/J mice with post-operative cognitive dysfunction R—ketamine (10 mg/kg, i.p.), TGF-β1 inhibitor RepSox (10 mg/kg, i.p.) for 14 days (Zhu et al., 2025)	Black-Gold II staining Mbp immunofluorescence	R-ketamine restores the level of Mbp immunofluorescence and the density of Black-Gold II staining in the corpus callosum. Pre-treatment with the TGF-β1 inhibitor RepSox blocks the pro-myelinating effects of R-ketamine	R-ketamine promotes axon remyelination in the corpus callosum following surgery at least partially through TGF-β1 signaling
Adult male C57BL6/J mice, treated with cuprizone food pellets R—ketamine (10 mg/kg, i.p.), TGF-β1 inhibitor RepSox (10 mg/kg, i.p.) for 14 days (Zhao et al., 2024)	Black-Gold II staining Mbp immunofluorescence	R-ketamine restores the level of Mbp immunofluorescence and the density of Black-Gold II staining in the corpus callosum. Pre-treatment with the TGF-β1 inhibitor RepSox blocks the pro-myelinating effects of R-ketamine	TGF-β1 signaling might be involved in R-ketamine’s pro-myelinating effects in the corpus callosum in a model of demyelination-remyelination
Adult female C57BL6/J mice, immunized with myelin oligodendrocyte glycoprotein (MOG)_35–55_ and killed *Mycobacterium tuberculosis* [model of experimental autoimmune encephalomyelitis] R-ketamine (10 mg/kg/day) daily from 30 min before the injection of emulsions to the 14th day (Wang et al., 2021)	Histopathology Immunofluoresecnce	(R)-ketamine attenuated pathological score in the spinal cord compared to control (R)-ketamine ameliorated the area of demyelination in the spinal cord compared to control	Ketamine might elicit protective effects on WM in effects in autoimmune myelin-degenerative disorders
Adult male C57BL/6 J mice, treated with cuprizone food pellets R-ketamine (10 mg/kg, i.p.) (Wang et al., 2022)	Fecal 16S rRNA sequencing SCFA determination (HPLC-CDD) Histopathology Mbp and ionized calcium-binding adaptor molecule 1 Iba-1 Immunofluorescence	The genera *Eisenbergiella* and *Mahiella* are increased by cuprizone and normalized by ketamine Ketamine reversed the cuprizone-induced increases in *Eisenbergiella massiliensis* and *Butyrivibrio proteoclasticus* and the decrease in *Clostridium bolteae* *Eisenbergiella massiliensis* abundance positively correlated with demyelination and Iba1 immunoreactivity and negatively correlated with fecal lactic acid, which ketamine restored Pro-inflammatory taxa *Butyrivibrio proteoclasticus*, *Faecalibaculum rodentium*, and *Bacteroides sartorii* positively correlated with Iba1 immunoreactivity, while *Lactobacillus murinus*, known for anti-inflammatory effects, negatively correlated with Iba1 immunoreactivity	RAADs may indirectly promote myelin repair through protective effects on the GBA and resulting effects on microglial activity
Adult male DBA/2 J mice Ketamine (10 mg/kg, i.p.) (Herzog et al., 2021)	Proteomics (LC-ESI-MS/MS) CSF (cerebrospinal fluid) proteomics	Mbp, myelin proteolipid protein (Plp1), signaling protein 14–3-3 protein sigma (Sfn), and myelinotrophic and neurotrophic factor prosaposin (Psap) are downregulated 4 h after ketamine	Acute CSF proteomics changes following ketamine suggests that myelination processes are significantly affected
Male adult C57BL/6 mice R—ketamine (10 mg/kg, i.p.), TGF-β1 inhibitor RepSox (10 mg/kg, i. p.) once after the end of chronic restraint stress Chronic restraint stress for 14 days (Xu et al., 2024)	Immunostaining Black-Gold II staining Mbp expression Myelin thickness	R-ketamine alleviates the chronic restraint stress-induced demyelination and reduced Mbp immunoreactivity in the corpus callosum The size of demyelination area is positively correlated with depressive-like behavior The beneficial effects of R-ketamine on myelination in the corpus callosum are blocked by pretreatment with the TGF-β1 inhibitor RepSox	R-ketamine ameliorates stress-induced demyelination in the corpus callosum, and increases Mbp levels putatively through a mechanism that involves TGF-β1 signaling
(Huang et al., 2023) Adult male C57BL6/J mice Chronic social defeat stress Ketamine, S-ketamine, R-ketamine (10 mg/kg, i.p.) Cortical OPCs culture	Spatial transcriptomics Transmission electron microscopy Immunohistochemistry Luxol fast blue staining Western blotting *In vivo* two-photon and calcium imaging	Restored Mbp, Plp and Mobp 7 days after a single administration of ketamine in the medial PFC and hippocampus. Restored decreased myelin sheath thickness and length, and the reduced number of myelinated axons in the brain by promoting the differentiation of OPCs into mature OLs through an α-amino-3-hydroxy-5-methyl-4-isoxazolepropionic acid receptor (AMPA) receptor-mediated mechanism. Inhibition of the expression of MOBP blocked ketamine’s long-lasting antidepressant effects.	Ketamine can restore myelin integrity in stress-compromised circuits by promoting OPC differentiation and increasing expression of key myelin genes (Mbp, Plp, Mobp). Activity-dependent myelin remodeling appears to be a critical component of ketamine’s sustained antidepressant effects,
Adult male C57BL6/J mice undergoing Brachial plexus root avulsion (BPRA) and replantation model S—ketamine (10 mg/kg, i.p.) (Huang et al., 2025)	Electron microscopy G-ratio (inner/outer diameter of the myelin sheath)	S-ketamine restored the BPRA-induced decreased G-ratio of the myocutaneous nerve	S-ketamine promotes axon remyelination following nerve injury
Landrace-Large-White swine of both sexes Ketamine (12.5, 25 and 500 mg, subarachnoid) Ketamine (12.5, 25 and 50 mg/day, subarachnoid) for 7 days (Errando et al., 1999)	Histopathologic examination (light microscopy) with hematoxylin–eosin or Luxol-fast blue myelin staining Myelination	No myelin alterations observed	Repeated subarachnoid ketamine administration does not appear to elicit myelin toxicity in swine
Male adult C57BL/6 mice S-ketamine (3 mg/kg, i.p.) (Weckmann et al., 2018)	Mbp protein level in the hippocampus (Western blot and proteomics)	S-ketamine increases hippocampal Mbp levels 2 and 24 h after a single injection and this increase in inversely proportional to the time spent floating in the forced swim test	The ketamine-induced increase in Mbp might be related (i.e., a cause or consequence) to the antidepressant effects observed
Large myelinated fibres from the sciatic nerve of the clawed toad *Xenopus laevis* Ketamine (50 mg/mL) (Arhem and Rydqvist, 1986)	Voltage clamp	Ketamine application reversibly decreases sodium and potassium currents associated with a potential step At low concentrations, only the sodium currents are decreased A decrease in peak sodium current amplitude and a dose-dependent positive shift of the curve is observed upon adding ketamine to the medium Ketamine application reversibly decreases the potassium current, yet to a lesser extent compared to the sodium current	Ketamine transiently modulates axonal excitability, which might influence myelin plasticity and WM function
Studies with higher-than therapeutic regimens			
Male and female ketamine-dependent subjects and 44 age-matched healthy volunteers No intervention, observational study (Liao et al., 2010)	Diffusion MRI	Individuals with ketamine dependence display bilateral frontal and left temporoparietal reductions in FA. Values in the left and right WM, but not the left temporoparietal WM, negatively correlated with the total lifetime ketamine consumption	Ketamine abuse elicits negative outcomes on myelination in frontal and temporoparietal brain areas
Individuals with ketamine addiction No intervention, observational study (Wang et al., 2013)	Diffusion MRI	Early degeneration in the superficial WM of the cortex, appearing as hyperintense lesions as early as 1 year and evolving in to larger sites of atrophy after 4 years, indicating early myelin damage	Ketamine abuse leads to myelin damage
Male and female adult ketamine users and polydrug users No intervention, observational study (Edward Roberts et al., 2014)	Structural and diffusion MRI	Reduced axial diffusivity (AD) in ketamine abusers is observed mainly in prefrontal and parietal areas	Chronic ketamine use is associated with WM disruptions in frontoparietal networks
Individuals with ketamine abuse, individuals with poly-drug use including ketamine No intervention, observational study (Liang et al., 2020)	Diffusion MRI	Poly-drug users using ketamine had larger total WM volumes than both controls and primarily ketamine users Larger WM volumes were associated with better verbal learning, earlier onset of ketamine use, and greater drug dependence severity	Baseline WM integrity and post-treatment changes may predict and influence ketamine’s antidepressant effects
Male and female chronic ketamine users No intervention, observational study (Chesters et al., 2022)	Volumetric MRI	Cerebellar WM and total cerebral WM did not differ between groups	Chronic ketamine use might not damage WM
Male adolescent Cynomolgus Monkeys Ketamine (1 mg/kg, i.v.) daily for 3 months (Li et al., 2017)	Diffusion MRI	Significantly decreased FA in the right side of sagittal striatum, posterior thalamic radiation, middle temporal gyrus, inferior frontal gyrus. Retrolenticular limb of the internal capsule and superior longitudinal fasciculus	Chronic adolescent ketamine exposure disrupts WM microstructure in several brain regions
Male and female juvenile rhesus macaques (12 and 18 months old) Ketamine (10 mg/kg, i.m.), several times a year (Young et al., 2021)	Diffusion MRI	Higher rates of exposure to ketamine is associated with WM microstructural abnormalities such as lower FA and higher AD, MD and RD	Repeated ketamine exposure during youth is linked to detrimental changes in WM microstructure
Postnatal day 5 (P5) Sprague–Dawley rat pups of both sexes Ketamine (50 mg/kg, i.p.) for 5 days (Zhang et al., 2019)	Mbp immunofluorescence	Mbp immunofluorescence is increased in the mPFC (but not the hippocampus) of female rats receiving neonatal ketamine administration Mbp density in the CA1 region of the hippocampus correlates positively with path length in the Morris Water Maze	Neonatal repeated high dose ketamine leads to overmyelination in the mPFC in female but not male rats, which is correlated with learning dysfunction
Male and female Sprague–Dawley rat pups S-ketamine (40 mg/kg, i.p.) three times per day at 2 h intervals for 3 days (Liu et al., 2024)	Histopathology Immunofluorescence Western blot	S-ketamine exposure reduces Mbp expression in the corpus callosum (CC) and reduces CC1 + OLs S-ketamine exposure increased the absolute number of Cas3 + CC1 + OLs S-ketamine exposure significantly increased the number of PDFGRα+ OPCs S-ketamine exposure significantly decreased O4 + OLs Progesterone ameliorated the S-ketamine-induced hypomyelination via the PI3K/Akt signaling pathway	Ketamine during the perinatal period disrupts OL maturation and reduces myelin-related protein expression in the developing brain
Neonatal male rats (strain not specified) S-ketamine (25 or 50 mg/kg, i.p.) for 3 days (Zhou et al., 2025)	Diffusion MRI Luxol fast blue myelin staining	S-ketamine leads to long-term (2 weeks after) changes in water diffusivity in the anterior and posterior regions of the CC ADC values following S-ketamine are lower in the CC splenium and right cingulate AD values are significantly lower and RD significantly higher following lower and higher dose S-ketamine ADC and RD values in the corpus callosum (CC) splenium are markedly lower in the high-dose S-ketamine group The high-dose group shows a marked decrease in AD values in the CC splenium compared to controls In the anterior CC, relative to the controls, the isotropy of the splenium of the CC is markedly increased in the high-dose S-ketamine group The isotropy of the left cingulum is markedly increased in the high-dose group In the posterior CC, compared with the control and low-dose groups, isotropy of the CC splenium is significantly higher in the high-dose S-ketamine group	Repeated high-dose neonatal exposure to S-ketamine leads to persistent and complex alterations in the microstructure of WM, affecting both myelin and axonal components, which may contribute to behavioral abnormalities, motor impairments, and deficits in spatial memory
Neonatal rats S-ketamine (40 mg/kg, i.p.) 3 times a day at 2 h intervals. Triiodothyronine (T3, 0.1 μg/kg, i.p., 10 min after S-ketamine) Peroxisome Proliferator-Activated Receptor Alpha (PPARα) inhibitor GW6471 (2 mg/kg, i.p., 1 h prior to S-ketamine) Fenofibrate (100 mg/kg, i.p., 1 h prior to S-ketamine) OLN-93 OL cell line 900 μM S-ketamine, and 900 μM S-ketamine combined with 50 nM triiodothyronine (T3, thyroid replacement therapy) for 24 h (Shan et al., 2025)	Western blot Transmission electron microscopy Immunofluorescence staining Metabolomics (MS/MS) Mbp protein level in the corpus callosum and cerebellum Myelin thickness G-ratio Proliferation and differentiation of OPCs Lipid metabolomics	Mbp protein level in the corpus callosum and cerebellum and Mbp fluorescence intensity in the corpus callosum are significantly reduced following S-ketamine. These effects are mitigated by treatment with T3 The thickness of corpus callosum myelin sheaths in S-ketamine-treated rats is lower and the G-ratio is higher compared to controls. These effects are mitigated by treatment with T3 The percentage of proliferating OPCs is decreased following S-ketamine treatment, and T3 does not reverse this decrease The number and percentage of mature OLs decreases following treatment with S-ketamine. Treatment with T3 increase the proportion of mature OLs Enrichment analysis of differentially expressed metabolites showed that the top canonical pathways were related to glycerophospholipids metabolism. Many metabolites of the glycerophospholipid metabolic pathway were significantly affected PPARα expression in OPCs is reduced by S-ketamine, and this effect is mitigated by T3 treatment. The PPARα inhibitor GW6471 inhibits OPC differentiation in the S-ketamine/T3 treated rats Fenofibrate partially reverses the ketamine-induced impaired maturation of OPCs	Neonatal S-ketamine-induced hypomyelination is mitigated by T3 treatment at least partially by reducing the inhibitory effect of S-ketamine on the differentiation and maturation of OPCs Inhibition of PPARα signaling by S-ketamine might be involved in the impaired maturation of OPCs
*In vitro* co-cultures of human neural cell types, including hTERT-immortalized fetal microglia and astrocytes, primary human neurons, and OPCs High-dose ketamine (25–150 μM) for 6 h (Penning et al., 2021)	Cell viability (LDH, Live/Dead assays) Proliferation (ViaLight) Activation markers (PCR, WB, ELISA), Apoptosis (caspase 3 assay, WB) Cytokine secretion (ELISA) Phagocytosis assay EV isolation and characterization Gene silencing EV secretion inhibition	Ketamine inhibits cell proliferation and induces dose-dependent cell death and caspase-3 activity in OPCs	Ketamine’s neurotoxic effects at high doses involve complex neuron–glia interactions and BDNF pathway disruption
Embryonic neural stem cells from embryonic/gestational day 16 Sprague–Dawley rats Ketamine (10 μM) for 24 h (Slikker et al., 2015)	O4 immunostaining	No changes in the number of O4-positive OLs in ketamine-exposed cultures	Ketamine does not affect the number of OLs *in vitro*

The table details model characteristics, interventions, experimental methods, parameters evaluated, key results, and main conclusions. Studies are grouped by compound class (ketamine and serotonergic psychedelics) and dose range (therapeutic vs. higher-than-therapeutic). Findings collectively highlight the bidirectional effects of ketamine these rapid-acting antidepressants (RAADs) on WM microstructure and OL-related processes, suggesting potential mechanisms of neuroplasticity or toxicity depending on dose, duration, and developmental stage. OL, oligodendrocyte; OPC, oligodendrocyte precursor cell; WM, white matter; TRD, treatment-resistant depression; MDD, major depressive disorder; MRI, magnetic resonance imaging; FA, fractional anisotropy; MD, mean diffusivity; RD, radial diffusivity; AD, axial diffusivity; NDI, neurite density index; FD, fiber density; MBP, myelin basic protein; NF200, neurofilament 200 kDa; MRS, magnetic resonance spectroscopy; MOG, myelin oligodendrocyte glycoprotein; TGF-β1, transforming growth factor beta 1; PAG, periaqueductal gray; mPFC, medial prefrontal cortex; PCC, posterior cingulate cortex; PCu, precuneus; OLN, oligodendrocyte-like neural cell line; PPARα, peroxisome proliferator-activated receptor alpha; T3, triiodothyronine; GW6471, PPARα inhibitor; ADC, apparent diffusion coefficient; CSF, cerebrospinal fluid; DRN, dorsal raphe nucleus; EF, encephalitogenic factor; MBP, myelin basic protein; 5-HT, serotonin (5-hydroxytryptamine); RAAD, rapid-acting antidepressant; LSD, lysergic acid diethylamide; DMT, N, N-dimethyltryptamine; 5-MeO-DMT, 5-methoxy-N, N-dimethyltryptamine; 5-Cl-DMT, 5-chloro-N, N-dimethyltryptamine; DOM, 2,5-dimethoxy-4-methylamphetamine; DOI, 2,5-dimethoxy-4-iodoamphetamine; c-Fos, immediate early gene marker; MBP, myelin basic protein; G-ratio, ratio of inner to outer axonal diameter; HPLC-CDD, high-performance liquid chromatography with charged aerosol detector; LC-ESI-MS/MS, liquid chromatography–electrospray ionization tandem mass spectrometry; MRS, magnetic resonance spectroscopy; EAE, experimental autoimmune encephalomyelitis; EF, encephalitogenic factor; Sfn, 14-3-3 sigma protein; Plp1, myelin proteolipid protein; Psap, prosaposin; TBSS, tract-based spatial statistics; OLs, oligodendrocytes; SCFA, short-chain fatty acids; BDNF, brain-derived neurotrophic factor.

**Table 2:** Summary of experimental and clinical studies investigating the effects of serotonergic psychedelics on white matter (WM) structure, myelination, and oligodendrocyte (OL) biology.

Model and characteristics Intervention Reference	Experimental methods Parameters evaluated	Results	Significance
Serotonergic psychedelics			
Microsomal-myelin layer in homogenates from squirrel monkey brain sections LSD (0.5, 1, and 2 mg/kg, i.v.) (Snyder and Reivich, 1966)	Spectophotofluorimetry to determine LSD concentration in target areas/cellular fractions	About 1/5th of the LSD detected in the brain is contained in the microsome-myelin fraction following separation of brain homogenates	Historical evidence- LSD might bind directly to myelin
Adult male Charles River CD rats LSD (0.2, 2, or 10 mg/kg, i.p.) (Faragalla, 1972)	Spectrophotofluorometric assay to determine the LSD content in the myelin fraction of brain homogenates	5% of the LSD detected in brain homogenates subcellular fractions is detected in the myelin fraction Even at the highest dose, the subcellular myelin brain fraction does not appear to reach saturation	Historical evidence- LSD might interact with myelin or myelin-related proteins
Lymphocytes, macrophages, and encephalitogenic protein from the blood of patients with malignant neoplasia or organic disease of the nervous system LSD, 5-Cl-DMT, bufotenine, DMT, 5-MeO-DMT, 5-methoxytryptamine, mescaline, dimethyl DOM (130 μg/mL). (Carnegie et al., 1972)	Cytopherometer assay [early immunological technique to measure cell interactions] with lymphocytes, macrophages, and encephalitogenic protein from the blood of patients with malignant neoplasia or organic disease of the nervous system	LSD, 5-Cl-DMT, bufotenine, DMT, 5-MeO-DMT, 5-methoxytryptamine, mescaline, dimethyl DOM partially block (in decreasing order) the interaction between EF, (putatively MBP) and sensitized lymphocytes	Historical evidence- Serotonergic RAADs can mimic 5-HT ability to interact with MBP putatively through binding to its tryptophan-rich antigenic site
Butanol extracts from myelin fragments derived from brain areas of male adult Wistar rats (Ishitani et al., 1978b)	Competition of 5-HT binding to butanol extracts from myelin fragments	LSD does not inhibit 5-HT binding to myelin fragments	Historical evidence- LSD might not bind directly to myelin fragments
Theoretical study (Root-Bernstein, 1983).	Structural modeling, molecular interaction analysis (*π*–π stacking, hydrogen bonding, ionic interactions), and comparative interpretation of binding affinity data	LSD and 5-HT bind competitively to a specific site in MBP, located at residues Arg-Phe-Ser-Trp The model shows that LSD and 5-HT are “molecularly sandwiched” between aromatic residues via π–π stacking, hydrogen bonding, and ionic interactions	LSD might bind MBP in the brain
12 weeks old male Fischer 344 rats (+)-d-LSD (+)-tartrate (2:1) (0.1, 0.25, 0.5, 1.0 mg/kg, i.p.) (Reissig et al., 2008)	Immunocytochemistry Light, fluorescence, and confocal microscope analysis Forebrain cFOS+ cells (neurons, oligodendrocytes, astrocytes, microglia)	LSD induced c-Fos in 14% of Olig1 + OLs, but not in astrocytes or microglia	LSD induces activity-dependent gene expression not only in neurons but also in a subset of OLs, suggesting potential direct functional engagement
8–10-week-old male C57BL6/N mice LSD (30 μg/kg) per day per 7 days (Inserra et al., 2022)	Whole genome bisulfite sequencing of the mPFC	Increased promoter methylation of Yin Yang 1 (an OPC-specific repressor) in the mPFC, putatively reducing its expression	Epigenetic modulation by RAADs may enhance OPC differentiation and myelination through epigenetic mechanisms
Adolescent male and female C57BL6/J mice Single administration or 6 treatments over 2 weeks of 3.3 μg of LSD (p.o.) (Harris-Blum et al., 2024)	Diffusion MRI	Single dose LSD increases apparent diffusion coefficient (ADC) in the reticular thalamic nucleus, medial geniculate, CA3 region of the hippocampus, 4th cerebellar lobule, central medial thalamic nucleus, and CA1 region of the hippocampus, and it decreases it in the anterior cingulate nucleus, secondary motor cortex, and paragigantocellular nucleus Repeated LSD increases ADC in the vestibular nucleus, basal amygdaloid nucleus, dentate gyrus, central medial thalamic nucleus, periaqueductal gray, medial geniculate, parafascicular thalamic nucleus, parabrachial nucleus, globus pallidus, entorhinal cortex, posterior thalamic nucleus, lateral posterior thalamic nucleus, reticular thalamic nucleus, zona incerta, subiculum, caudate putamen, crus of ansiform lobule, pedunculopontine tegmental nucleus, external capsule, mesencephalic reticular formation, 5th cerebellar lobule, locus coeruleus, internal capsule, corpus callosum, ventral thalamic nucleus, solitary tract nucleus, anterior hypothalamic nucleus, lateral caudal hypothalamic nucleus, cerebellar nuclear nucleus, central amygdaloid nucleus, cerebral peduncle, inferior colliculus, bed nucleus stria terminalis, cuneate nucleus, lateral preoptic nucleus, paramedian lobule, anterior pretectal thalamic nucleus, auditory cortex, lateral rostral hypothalamic nucleus, 6th cerebellar lobule, dorsal hippocampal commissure, CA1 region of the hippocampus, dorsal raphe, accumbens core, stria terminalis, medial preoptic nucleus, medial amygdaloid nucleus, 7th cerebellar lobule, accumbens shell, anterior commissure, secondary somatosensory cortex, parvicellular reticular nucleus, 8th cerebellar lobule, lateral amygdaloid nucleus, 2nd cerebellar lobule, caudal piriform cortex, medial dorsal thalamic nucleus, lateral dorsal thalamic nucleus, lateral septal nucleus, lateral geniculate, forceps minor corpus callosum, superior colliculus, diagonal band of Broca, temporal cortex, reuniens thalamic nucleus, substantia nigra, extended amygdala, medial lemniscus, flocculus cerebellum, infralimbic cortex, 4th cerebellar lobule, primary somatosensory cortex, principal sensory nucleus trigeminal, CA3 Single dose LSD decreases FA in the lateral caudal hypothalamic nucleus, reticular thalamic nucleus, globus pallidus, lateral rostral hypothalamic nucleus, interpeduncular nucleus, tenia tecta cortex, and increases it in the temporal cortex, secondary motor cortex, 7th cerebellar lobule, stria terminalis, and habenular nucleus Repeated dose LSD decreases FA in the medial mammillary nucleus, lateral caudal hypothalamic nucleus, vestibular nucleus, CA1 region of the hippocampus, cerebellar nuclear nucleus, pedunculopontine tegmental nucleus, lateral posterior thalamic nucleus, 10th cerebellar lobule, medial lemniscus, principal sensory nucleus trigeminal, solitary tract nucleus, basal amygdaloid nucleus, parabrachial nucleus, locus coeruleus, pontine reticular nucleus oral, reticulotegmental nucleus, and posterior thalamic nucleus	LSD induces region-specific microstructural WM changes, with a single dose showing mixed effects on WM Repeated LSD administration leads to widespread ADC increases and FA decreases, suggesting potential disruption of myelin structure with sustained exposure during adolescence
Adult C57BL/6 J male mice, mice from the University of Wisconsin-Madison Breeding Core, mice from Jackson Laboratories Psilocybin (3 mg/kg, i.p.) (Frautschi et al., 2025)	*Ex-vivo* diffusion MRI	Psilocybin leads to higher mean tract length in the frontal association cortex 72 h but not 24 h following administration Psilocybin increases MD in the primary visual cortex 24 h following administration Psilocybin increases MD in the thalamus, hippocampus, and striatum 72 h following administration Psilocybin does not affect FA 24 h or 72 h after administration Psilocybin decreases neurite density index (NDI) in the hippocampus and striatum 72 h after administration	Psilocybin induces microstructural changes consistent with altered myelination in regions implicated in mood regulation. Psilocybin may promote adaptive myelin remodeling
OL lineage cells (OPCs to mature OLs) from P1 rat forebrain; neuron-OL co-cultures from E16 rat spinal cord; characterized by stage-specific markers (NG2, O4, O1, MBP) DOI (0.1–10 μM); 5-HT (1, 5, 25, 100 μM); with or without pre-treatment of 5-HT_2A_ antagonist ritanserin (1 μM) (Fan et al., 2015)	Cell Death ELISA, cleaved caspase-3 expression Percentage of cell death relative to control	DOI exposure at 1, 5, 25, and 100 μM caused dose-dependent cell death in OPCs and immature OLs, with immature OLs showing greater vulnerability. Ritanserin pre-treatment reduced DOI-induced apoptosis	DOI activation of 5-HT2A receptors reveals a vulnerability of immature OL to receptor-mediated cytotoxicity

The table details model characteristics, interventions, experimental methods, parameters evaluated, key results, and main conclusions. Findings collectively highlight the bidirectional effects of serotonergic psychedelics on WM microstructure and OL-related processes, suggesting potential mechanisms of neuroplasticity or toxicity depending on dose, duration, and developmental stage. OL, oligodendrocyte; OPC, oligodendrocyte precursor cell; OLs, oligodendrocytes; WM, white matter; MRI, magnetic resonance imaging; ADC, apparent diffusion coefficient; FA, fractional anisotropy; MD, mean diffusivity; NDI, neurite density index; MBP, myelin basic protein; mPFC, medial prefrontal cortex; 5-HT, serotonin (5-hydroxytryptamine); RAAD, rapid-acting antidepressant; LSD, lysergic acid diethylamide; DMT, *N,N*-dimethyltryptamine; 5-MeO-DMT, 5-methoxy-*N,N*-dimethyltryptamine; DOI, 2,5-dimethoxy-4-iodoamphetamine; c-Fos, immediate early gene marker.

### 3.2 Ketamine

Ketamine was the most frequently studied compound in the dataset, with evidence spanning human, animal, and *in vitro* models. Ketamine treatment exhibits dose-dependent and context-specific effects on brain structure and function, with low or therapeutic doses generally producing neuroprotective and pro-myelinating benefits, while high or repeated doses generally induce neurotoxic and demyelinating changes.

#### 3.2.1 Therapeutic doses

Consistent with its dose-dependent profile, at low or therapeutic regimens (generally one or few administrations of up to 10 mg/kg), ketamine exerts beneficial neuroplastic and pro-myelinating effects, some of which may predict, and correlate with, clinical responsiveness. Diffusion tensor imaging (DTI) in individuals with major depression showed that a single administration of ketamine increased fractional anisotropy (FA, suggesting increased myelin density) in the cingulum of responders compared to non-responders (Vasavada et al., 2016). A recent study in individuals with treatment-resistant depression (TRD) found that higher pre-infusion FA in the left cingulum bundle and left superior longitudinal fasciculus was significantly associated with greater improvement in depressive symptoms 24 h after infusion. Conversely, larger post-ketamine increases in FA were linked to poorer symptom improvement, particularly in the uncinate fasciculus and forceps minor (Sydnor et al., 2020). Consistently, another study identified the left cingulum bundle as a region where higher baseline tissue-specific FA predicted clinical response to ketamine in individuals with TRD (Langhein et al., 2022). Complementing these findings, repeated ketamine administration to individuals with TRD leads to microstructural changes reflected by a decrease in the MRI-derived neurite density index (NDI) in key WM tracts, with such remodeling correlating with clinical improvements, particularly in anhedonia (Taraku et al., 2023). In contrast, a study using combined S-ketamine and escalating doses of sertraline found no reversal of widespread WM abnormalities in individuals with MDD (Liu et al., 2025). Lower pre-treatment fiber density within medial Prefrontal Cortex—Precuneus/Posterior Cingulate Cortex (mPFC-PCu/PCC) and mPFC-PAG (periaqueductal grey) pathways correlated with greater pain relief after repeated individualized ketamine infusion followed by oral ketamine (Mills et al., 2024).

In the implantation mouse model of peripheral nerve injury, which induces demyelination reflected by an increased G-ratio (i.e., thinner myelin) of myocutaneous nerve axons in the brachial plexus, S-ketamine treatment restored the G-ratio to values comparable to those of controls, indicating a remyelinating or myelin-protective effect (Huang et al., 2025). In a mouse model of postoperative cognitive dysfunction, repeated R-ketamine for 14 days promoted corpus callosum remyelination as measured by increased myelin basic protein (Mbp) immunofluorescence and density of Black-Gold II staining (Zhu et al., 2025). Interestingly, pre-treatment with the TGF-β1 inhibitor RepSox blocked the pro-myelinating effects of R-ketamine (Zhu et al., 2025). In an experimental autoimmune encephalomyelitis model, which mimics inflammatory demyelination observed in multiple sclerosis, adult female C57BL/6 J mice were immunized with myelin oligodendrocyte glycoprotein (MOG_₃₅–₅₅_) and killed *Mycobacterium tuberculosis* and treated daily with R-ketamine from 30 min before immunization to day 14 (Wang et al., 2021). Histopathological and immunofluorescence analyses showed that ketamine significantly attenuated spinal cord pathology and markedly reduced the demyelinated area compared to saline-treated mice (Wang et al., 2021). Supporting a role for TGF-β1 signaling in the pro-myelinating effects of therapeutic doses of ketamine, the authors in another study confirmed that the beneficial effects of R-ketamine on remyelination in the brain of cuprizone-treated mice were blocked by the TGF-β1 inhibitor RepSox (Zhao et al., 2024). Further corroborating these findings, adult male mice exposed to chronic restraint stress receiving a single dose of R-ketamine displayed alleviated stress-induced demyelination in the corpus callosum and increased Mbp expression, as confirmed by immunostaining and Black-Gold II labeling. These beneficial effects of R-ketamine were abolished by pre-treatment with the TGF-β1 inhibitor RepSox. Notably, the extent of demyelination was positively correlated with the severity of depressive-like behaviors (Xu et al., 2024).

In rats, a single injection of ketamine (25 mg/kg) increased FA and decreased RD in the dorsal raphe nucleus (DRN), the ventral hippocampus, the infralimbic (IL-) PFC and corpus callosum in both hemispheres, together with the amygdala, nucleus accumbens (NAc), and orbifrontal cortex only in the right hemisphere. The rise in FA was initially observed in the DRN and right NAc 24 h after drug administration, and it persisted after 1 week, while the effect in other brain regions innervated by 5-HT neurons was noted 1 week after ketamine administration (Pascual-Antón et al., 2021). Another study in rats reported that neonatal ketamine exposure increased Mbp density in the medial (m)PFC during adulthood in females, but not males (Zhang et al., 2019) In a mouse cerebrospinal fluid (CSF) proteomics study, therapeutic doses of ketamine acutely downregulated Mbp, myelin proteolipid protein (Plp)1, 14–3-3 protein sigma (Sfn), and prosaposin (Psap), and the associated biological function, while 1 week later Mbp and Plp1 were upregulated (Herzog et al., 2021). Similarly, hippocampal Mbp levels were reported to be increased after 2 h and 24 h in mice, and this increase was directly proportional to the antidepressant-like effects elicited (Weckmann et al., 2018). Another study reported increased Mbp in the rat IL-PFC following a therapeutic doses ketamine (Pascual-Antón et al., 2021). Recent work suggests that the pro-myelinating effects of ketamine through myelin-associated OL-basic protein (Mobp)-dependent myelin might be required for its sustained antidepressant effects (Huang et al., 2023). In mice subjected to chronic stress, restored expression of the myelin-related genes Mbp, Plp, and Mobp was observed in the mPFC and hippocampus, 7 days after a single administration of ketamine. Ketamine restored the stress-induced decrease in myelin sheath thickness and length, and the reduced number of myelinated axons by promoting the differentiation of OPCs into mature OLs through an *α*-amino-3-hydroxy-5-methyl-4-isoxazolepropionic acid receptor (AMPA) receptor-mediated mechanism (Huang et al., 2023). Importantly, inhibition of the expression of Mobp blocked ketamine’s long-lasting antidepressant effects (Huang et al., 2023).

In a cuprizone-induced model of demyelination, ketamine reversed several gut-brain axis (GBA) alterations, affecting bacterial taxa composition and lactic acid—a short-chain fatty acid (SCFA)—production and metabolite of anaerobic pathways. Cuprizone increased the abundance of the phylum *Proteobacteria*, which ketamine restored to baseline levels. Similarly, the abundance of the genera *Eisenbergiella* and *Mahiella* (both belonging to the order *Clostridiales*) was increased by cuprizone but normalized by ketamine. At the species level, ketamine reversed the cuprizone-induced increases in *Eisenbergiella massiliensis* and *Butyrivibrio proteoclasticus,* as well as the decrease in *Clostridium bolteae.* Particularly, *E. massiliensis* abundance positively correlated with demyelination, ionized calcium-binding adaptor molecule 1 (Iba1) immunoreactivity and reduced fecal lactic acid, which ketamine restored. Importantly, this shift in fecal lactic acid production suggests potential functional changes in the gut metabolome. Positive correlations were also found between other pro-inflammatory taxa (*Butyrivibrio proteoclasticus, Faecalibaculum rodentium, Bacteroides sartorii*) and Iba1 immunoreactivity, while *Lactobacillus murinus*, with known anti-inflammatory effects, showed a negative correlation. These findings suggest that ketamine’s neuroprotective actions may involve a selective modulation of gut microbiome (GM) profiles that are associated with changes in Iba1-immunopositivity in the brain, indicating phenotypic shifts in the microglial population through the GBA (Wang et al., 2022). Following cuprizone withdrawal, the observed ketamine-induced microbiome changes were accompanied by increased remyelination and attenuated microglial changes compared to controls, based on Mbp and Iba1 immunoreactivity, respectively (Wang et al., 2022).

#### 3.2.2 Higher doses/abuse

Importantly, the effects of ketamine on WM and myelination appear to be dose-dependent and context-specific, showing contrasting outcomes across studies. While at sub-anesthetic or therapeutic doses (generally below 10 mg/kg) ketamine has been shown to exert neuroprotective and pro-myelinating effects, higher (generally more than 10 mg/kg and up to 100 mg/kg) or repeated doses are associated with neurotoxic and demyelinating changes. Consistent with this detrimental profile, one study (Liao et al., 2010) identified significant reductions in FA in the bilateral frontal and left temporoparietal WM of individuals with chronic ketamine use compared to controls. FA in the frontal WM of both hemispheres showed a negative correlation with the total amount of ketamine used across the individual’s lifetime (Liao et al., 2010). Ketamine abusers also exhibit reduced axial diffusivity (AD) in right hemisphere WM pathways, predominantly beneath the PFC, indicating lateralized and region-specific microstructural damage to axons or their organization (Edward Roberts et al., 2014). Additionally, disrupted WM connectivity between the caudate nucleus and lateral PFC correlates with the severity of long-term dissociative symptoms, suggesting that chronic ketamine use impairs both structural integrity and functional organization of cortico-subcortical circuits involved in emotion regulation and reality perception (Edward Roberts et al., 2014).

In contrast, a study reported that ketamine users have increased total WM and caudate volumes compared to controls (Liang et al., 2020). Notably, ketamine users who also consumed stimulants exhibited even greater WM volumes. In line with this, the study found that ketamine polydrug users had larger total WM volumes than both controls and users who primarily used ketamine. Interestingly, larger WM volume was associated not only with apparently positive outcomes, such as better verbal learning performance, but also with negative behavioral traits, including earlier onset of ketamine use and greater severity of drug dependence (Liang et al., 2020). These findings suggest that such volumetric increases may represent neuroadaptive or compensatory mechanisms in response to chronic drug exposure.

Further adding to this complexity, Chesters et al. (2022) reported no significant differences in cerebellar or total cerebral WM between chronic ketamine users and controls; however, because males and females were analyzed together, potential sex-dependent effects may have been obscured (Chesters et al., 2022). Consistent with neurotoxic interpretations, ketamine administration in human infants led to a significant decrease in frontal WM metabolites, including choline and Glx/creatine levels (Bhutta et al., 2012), pointing to the early vulnerability of developing WM to ketamine exposure.

Animal studies broadly support these clinical findings but further underscore the dose- and age-dependent nature of ketamine’s effects. Chronic ketamine exposure in adolescent *Cynomolgus* monkeys led to WM microstructural abnormalities, including reduced FA in the right side of sagittal striatum, posterior thalamic radiation (PTR), retrolenticular limb of the internal capsule (RLIC) and superior longitudinal fasciculus (SLF), and on the left side of PTR, middle temporal gyrus and inferior frontal gyrus. Diminished WM integrity was found in either fronto-thalamo-temporal or striato-thalamic connections with tracts including the SLF, PTR, and RLIC (Li et al., 2017). Consistently, higher rates of S-ketamine exposure in juvenile rhesus macaques was associated with widespread WM abnormalities, including lower FA and higher AD, MD, and RD (Young et al., 2021).

In neonatal rats, S-ketamine significantly reduced MBP protein levels and MBP fluorescence intensity in the corpus callosum and cerebellum, suggesting hypomyelination and compromised myelin structure. Indeed, myelin sheath thickness was reduced and the G-ratio increased (Shan et al., 2025). These structural deficits were mitigated by triiodothyronine (T3) treatment, which promoted OL maturation and partially restored MBP levels. Mechanistically, S-ketamine downregulated PPARα in OPCs, inhibiting their differentiation—an effect reversed by T3 and partially by the PPARα agonist fenofibrate. Complementary metabolomic data revealed dysregulation in glycerophospholipid metabolism, a pathway essential for membrane synthesis and myelin formation (Shan et al., 2025). Repeated high-dose exposure to S-ketamine in neonatal rats resulted in sustained and complex alterations in WM microstructure, particularly in the corpus callosum. Two weeks after treatment, brain imaging using DTI revealed significant changes in water molecule movement within the WM. Specifically, reductions in AD and increases in RD were observed, indicating compromised axonal structure and disrupted myelin integrity. These alterations were most pronounced in the anterior and posterior portions of the corpus callosum, including the splenium and right cingulate region. Histological analyses confirmed changes in WM structure, showing increased directional uniformity (isotropy) in both the anterior and posterior corpus callosum of rats exposed to the higher dose of S-ketamine. Importantly, these structural changes were associated with persistent impairments in behavior, particularly motor coordination and spatial memory (Zhou et al., 2025).

Complementing these findings, repeated neonatal ketamine administration led to sex-specific WM alterations, particularly in female Sprague–Dawley rats. Immunofluorescence analyses revealed increased MBP density in the mPFC, indicating over myelination, a pattern absent in male counterparts. Moreover, elevated MBP levels in the hippocampal CA1 region positively correlated with increased path length in the Morris Water Maze, suggesting a link between aberrant myelination and learning deficits (Zhang et al., 2019).

Protective interventions have also been identified: S-ketamine-induced hypomyelination in the developing rat brain, marked by reduced MBP and OL loss in the corpus callosum, was ameliorated by progesterone, acting via the PI3K/Akt signaling (Liu et al., 2024). Consistent with this, repeated neonatal high-dose ketamine similarly reduced Mbp levels and OLs numbers in the corpus callosum in rats, along with decreased OPC proliferation and differentiation, accompanied by PI3K/Akt dephosphorylation—a key signaling pathway involved in cell survival and growth (Liu et al., 2024). A similar study also identified decreased myelination and Mbp expression in the corpus callosum and cerebellum of neonatal rats, which could be ameliorated by T3 (Shan et al., 2025).

In contrast to these preclinical findings, an earlier study in swine reported no evidence of myelin toxicity following repeated subarachnoid ketamine administration (Errando et al., 1999). However, the study was limited by small sample size and relied on histopathologic examination with hematoxylin–eosin and Luxol fast blue staining, which may have lacked the sensitivity to detect subtle or early-stage WM changes. Notably, ketamine also modulates electrophysiological properties of myelinated fibers, as it reduces sodium and potassium currents in myelinated axons, with a greater effect on sodium currents (Arhem and Rydqvist, 1986).

These *in vivo* observations are supported by *in vitro* studies. *In vitro*, ketamine at doses equivalent to higher-than-therapeutic, elicited apoptosis in OPCs, and stimulated a pro-inflammatory response in astrocytes and microglia, which exacerbated ketamine’s neurotoxic effects on neurons (Penning et al., 2021). Contrastingly, exposure of embryonic neural stem cells to lower, clinically relevant doses produced no changes in OL generation (Slikker et al., 2015), reinforcing the notion that ketamine’s effects on oligodendrocyte lineage cells and WM are highly dependent on dose, developmental stage, and cellular context.

### 3.3 Serotonergic psychedelics

Concerning serotonergic RAADs (Table 2), no clinical studies were identified. *Ex vivo* diffusion MRI in psilocybin-treated adult mice revealed region-specific microstructural alterations, including increased tract length in the frontal association cortex and increased mean diffusivity in multiple limbic regions. Importantly, psilocybin also decreased neurite density in the hippocampus and striatum—areas implicated in mood regulation (Frautschi et al., 2025). DTI data from adolescent male and female mice showed that LSD induces widespread region-specific WM microstructural changes, with a single oral dose increasing apparent diffusion coefficient (ADC) in the reticular thalamic nucleus, medial geniculate, CA3 and CA1 regions of the hippocampus, 4th cerebellar lobule, and central medial thalamic nucleus, while decreasing it in the anterior cingulate nucleus, secondary motor cortex, and paragigantocellular nucleus (Harris-Blum et al., 2024). Repeated administration over 2 weeks led to broader ADC increases across more than 50 brain regions, including the vestibular nucleus, basal amygdaloid nucleus, dentate gyrus, globus pallidus, subiculum, entorhinal cortex, corpus callosum, external and internal capsules, locus coeruleus, auditory cortex, medial and lateral geniculate nuclei, substantia nigra, dorsal raphe, accumbens core and shell, stria terminalis, anterior commissure, and primary and secondary somatosensory cortices. Corresponding FA decreases were observed in many of these regions, notably the medial mammillary nucleus, cerebellar lobules, pedunculopontine tegmental nucleus, and CA1 of the hippocampus (Harris-Blum et al., 2024).

Historical empirical evidence shows that myelin-containing fractions, and possibly synaptosomal fractions, from rat and squirrel monkey brain tissue contain exogenously administered LSD (Snyder and Reivich, 1966; Faragalla, 1972). However, conflicting reports are present as to whether LSD itself and/or LSD binding sites are present in myelin fractions from the brains of rats (Farrow and Van Vunakis, 1972, 1973). RAADs such as LSD were previously shown to compete with 5-HT both *in vitro* and *in vivo* for a Mbp binding site in rats, suggesting a potential physical interaction with myelin or myelin-related proteins, although other studies failed to replicate these findings (Farrow and Van Vunakis, 1972; Ishitani et al., 1978a; Root-Bernstein, 1983). These discrepancies could be due to methodological differences or to the different subcellular fractions employed (Farrow and Van Vunakis, 1972; Ishitani et al., 1978a; Root-Bernstein, 1983). LSD has also been shown to bind directly to MBP (Carnegie, 1971; Field et al., 1971; Carnegie et al., 1972; Ishitani et al., 1977; Ishitani et al., 1978a), a finding supported by structural modeling (Smythies et al., 1972; Root-Bernstein, 1983).

Additionally, several indole-derivative psychedelics including LSD, N, N-DMT, 5-Cl-DMT, 5-OH-DMT, 5-MeO-DMT, mescaline, and DOM, were found to reduce, and in some cases (e.g., high-dose 5-Cl-N, N-DMT), to reduce or abolish the *ex vivo* reactivity to MBP of leukocytes from individuals with neurodegenerative disorders (Carnegie et al., 1972). Beyond binding studies, serotonergic RAADs have been shown to impact OL lineage cells and myelination pathways. LSD increased cFOS expression in Olig1-immunopositive (+) OL lineage cells in mice—cells identified by the expression of the transcription factor Olig1, which is a marker of OL lineage commitment of the rat PFC, especially in layers III, IV, and V (Reissig et al., 2008). In mice, repeated LSD administration increased the mPFC methylation of the Yin Yang 1 promoter, a key OPC-specific transcriptional repressor, thereby putatively removing inhibition on myelin-related gene expression and potentially enhancing myelination processes (He et al., 2007; Inserra et al., 2022).

Strengthening the dichotomous outcome of therapeutic vs. abuse doses, *in vitro* studies reported that prolonged exposure of rat-derived immature OLs and OPCs to DOI led to OPC and immature OL damage, an effect that was partially prevented by pre-treatment with the 5-HT_2A_ receptor-preferring antagonist ritanserin (Fan et al., 2015).

## 4 Discussion

**Figure 1:**
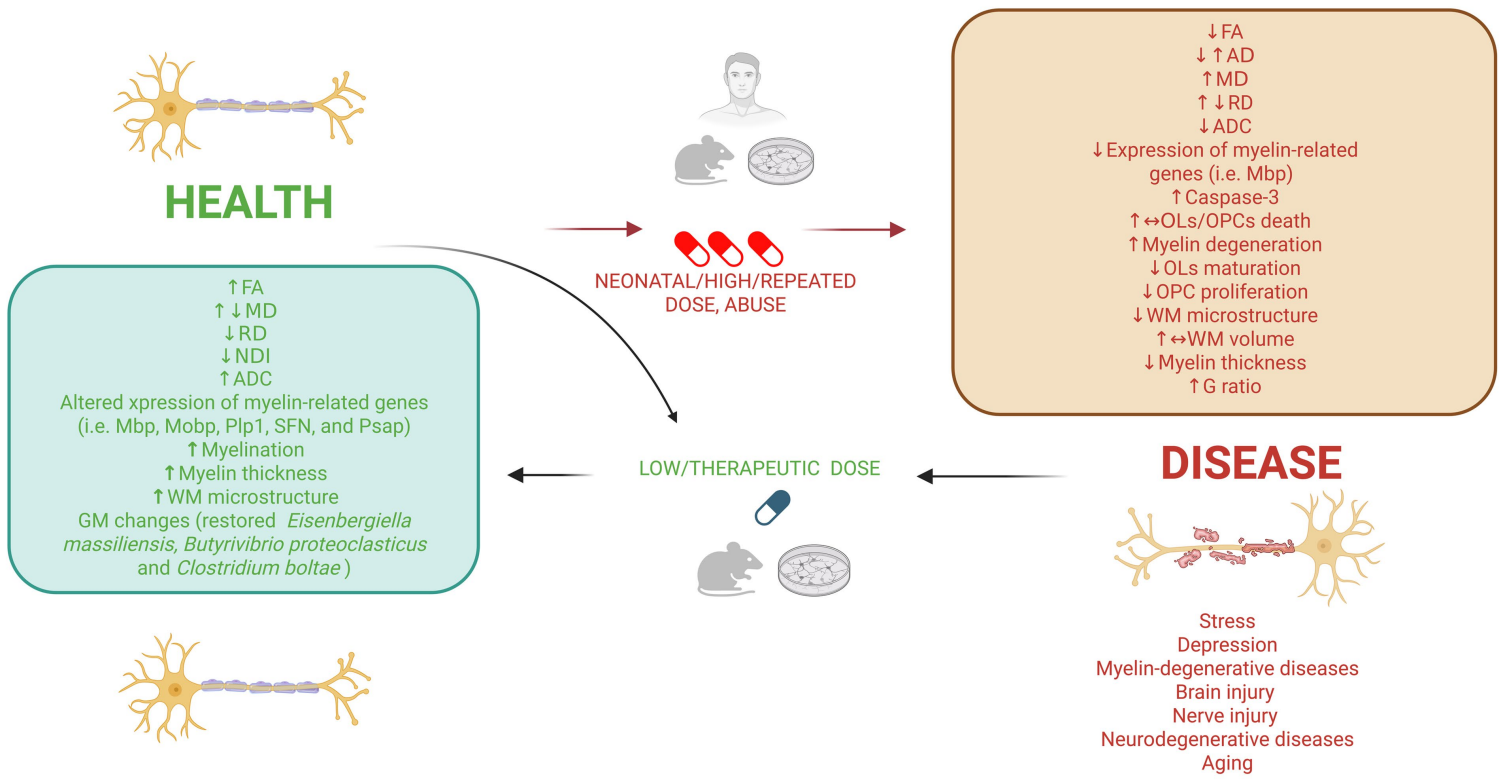
Dose- and context-dependent associations between rapid-acting antidepressant (RAAD) exposure and myelin-related outcomes across health and disease conditions. This figure summarizes how rapid-acting antidepressants (RAADs) affect myelin-related outcomes as a function of both dose and biological context, contrasting healthy or therapeutic conditions with those involving high-dose or repeated exposure. It also illustrates how RAADs may support myelin homeostasis in disease states, including stress, depression, neurodegeneration, aging, brain injury, and nerve injury. In disease or high-dose, repeated, or neonatal exposure conditions, RAADs are associated with reductions in fractional anisotropy (FA), increases in mean diffusivity (MD), radial diffusivity (RD), and apparent diffusion coefficient (ADC), as well as alterations in axial diffusivity (AD). These microstructural changes correspond to decreased expression of myelin-related genes such as Mbp (myelin basic protein), increased apoptotic signaling including caspase-3, impaired oligodendrocyte precursor cell (OPC) proliferation, reduced oligodendrocyte (OL) maturation, heightened OL/OPC vulnerability, myelin degeneration, decreased myelin thickness, and increased g-ratio. In contrast, under healthy or low-dose/therapeutic conditions, RAADs are associated with increases in FA, reductions in MD and RD, decreases in neurite density index (NDI), and increases in ADC, along with upregulation of myelin-related genes such as Mbp (myelin basic protein), Mobp (myelin-associated oligodendrocyte basic protein), Plp1 (proteolipid protein 1), Sfn (stratifin), and Psap (prosaposin). These changes are accompanied by increased myelination, greater myelin thickness, improved white-matter (WM) microstructure, and in one study, restoration of microbial taxa including *Eisenbergiella massiliensis*, *Butyrivibrio proteoclasticus*, and *Clostridium bolteae*. Overall, the figure highlights how RAAD effects on myelin biology vary according to dose, developmental stage, and health versus disease context. Created with BioRender.

This scoping review identified preliminary but increasingly converging evidence that RAADs influence myelin homeostasis through both direct and indirect mechanisms. Across 41 preclinical and clinical studies in humans, animals, and *in vitro* systems, RAADs were found to impact OLs, OPCs, and myelin-related proteins in a dose-, region-, and context-dependent manner. In addition to cell-intrinsic effects, changes in neuroimmune signaling and GBA communication also emerged as potential modulators of WM plasticity. Together, these findings suggest that alterations in myelin dynamics may contribute to both the therapeutic and adverse effects of RAADs, and warrant further investigation, as well as on mechanistic pathways and potential therapeutic target in neuropsychiatric disorders. Figure 1 provides a representation of the known effects and mechanisms involved.

### 4.1 Known NMDA-mediated effects on OLs and myelin

Glutamatergic signaling plays a central role in regulating OL/OPC development and myelination. Both NMDA and *α*-amino-3-hydroxy-5-methyl-4-isoxazolepropionic acid (AMPA) receptors are expressed on these cells, where their activation influences proliferation, migration, and differentiation (Traynelis et al., 2010). AMPA receptors are abundant during OPC maturation, while NMDA receptors exhibit high calcium permeability and modulate activity-dependent myelination. Although the precise contribution of the NMDA receptor to OPC maturation remains debated (De Biase et al., 2011), evidence suggests that cross-talk between NMDA and AMPA receptor signaling dynamically regulates OL function and myelin plasticity (De Biase et al., 2010; Spitzer et al., 2019). Given that ketamine primarily acts as an NMDA receptor antagonist while secondarily enhancing AMPA receptor throughput, it is well-positioned to modulate these processes, although direct receptor-specific evidence remains limited.

Ketamine was the most frequently studied compound in our dataset, spanning human, animal, and in vitro models, highlighting its complex and dose-dependent effects on myelination. OLs express NMDA receptors (Salter and Fern, 2005), suggesting that the NMDA receptor antagonist ketamine might directly affect myelination through signaling at these receptors on the principal cell types responsible for myelin production (Káradóttir et al., 2005). A seminal study demonstrated that OL lineage cells can engage in two distinct modes of myelination: one that is independent of neuronal activity and glutamate release, and another that relies on activity-dependent glutamate signaling (Lundgaard et al., 2014). Therefore, it cannot be excluded that ketamine might affect one or both of these modes of myelination in OLs. In this model, glutamate released during neuronal firing activates NMDA receptors on OL lineage cells, promoting myelination specifically of active axons. Furthermore, the protein neuregulin acts as a molecular switch that enhances NMDA receptor responses in OLs, biasing them toward this activity-dependent mode and accelerating overall myelin formation (Lundgaard et al., 2014). These mechanisms offer a mechanistic framework by which ketamine may modulate myelination through altering glutamatergic signaling pathways within OLs, and putatively OPCs. However, the precise downstream intracellular signaling cascades and cross-talk with other receptor systems involved in this process remain incompletely understood and warrant further investigation.

Importantly, ketamine is a racemic mixture composed of R-ketamine and S-ketamine. S-ketamine binds NMDARs with higher affinity and has greater anesthetic potency but more adverse effects, whereas R-ketamine exhibits more pronounced and sustained antidepressant effects in animal models (Zhang et al., 2014). Experimental studies indicate that R-ketamine more effectively repairs myelin damage and promotes OPC differentiation into mature oligodendrocytes compared to S-ketamine, with these effects dependent on AMPA receptor signaling (Huang et al., 2023). This suggests that the superior myelin-promoting and antidepressant effects of R-ketamine may arise from preferential activation of AMPA pathways, while S-ketamine exerts stronger NMDA antagonism.

At therapeutic doses, ketamine promotes myelin-related microstructural changes that appear to predict its antidepressant effects. Human imaging studies have shown that higher baseline FA in specific WM tracts, such as the cingulum, predicts better treatment response (Vasavada et al., 2016; Sydnor et al., 2020; Langhein et al., 2022), while changes in neurite density within WM tracts following repeated ketamine administration were significantly associated with clinical improvement, suggesting that ketamine may induce adaptive pruning of WM tracts, reflected by reduced apparent neurite density — a process that could relate to functional recovery mechanisms (Taraku et al., 2023). However, some interventions combining S-ketamine with other antidepressants have not consistently reversed WM abnormalities (Liu et al., 2025), suggesting variability across treatment modalities.

These findings highlight the complexity of ketamine’s effects on WM microstructure and suggest that individual patient factors, as well as polypharmacy, may modulate treatment outcomes. Future studies should carefully consider these variables to better understand how ketamine and related agents influence myelin integrity and to optimize personalized treatment strategies.

Human and non-human primate studies reveal that ketamine exposure can impact WM development and integrity, with infant data showing reduced choline and Glx/Cr ratios suggestive of impaired myelination (Bhutta et al., 2012), and adolescent non-human primates exhibiting decreased FA and disrupted WM microstructure after ketamine or S-ketamine treatment (Li et al., 2017; Young et al., 2021).

Preclinical rodent models further support a positive influence of therapeutic doses of ketamine on myelin, demonstrating increased FA and decreased radial diffusivity in mood-related brain regions following single ketamine doses (Pascual-Antón et al., 2021), as well as restoration of myelin ultrastructure and remyelination effects that depend on TGF-β1 signaling pathways (Huang et al., 2023; Xu et al., 2024; Zhao et al., 2024; Zhu et al., 2025). Additionally, ketamine was found to restore myelin integrity in a mouse model of nerve injury (Huang et al., 2025), and enhance myelin protein expression in mouse models of postoperative cognitive dysfunction (Zhu et al., 2025).

Collectively, these findings indicate that ketamine’s impact on myelin may vary across developmental stages and species but point toward a pro-myelinating mechanism mediated at least partially by TGF-β1 signaling. This underscores the importance of considering age, treatment timing, and molecular pathways when evaluating ketamine’s neurobiological effects and therapeutic potential.

Additionally, findings from an experimental autoimmune encephalomyelitis model further support the role of ketamine in protecting myelin under inflammatory conditions. In this paradigm, daily administration of R-ketamine attenuated spinal cord pathology and significantly reduced demyelination compared to saline-treated mice (Wang et al., 2021). These results indicate that R-ketamine not only promotes remyelination in toxin- or stress-induced models but also preserves myelin integrity in immune-mediated demyelination, suggesting potential therapeutic relevance for disorders, such as multiple sclerosis. Together with evidence implicating TGF-β1-dependent signaling in ketamine’s myelin-restorative effects, these data point to a broader capacity of ketamine to modulate neuroimmune–glial interactions that sustain WM homeostasis.

Mouse CSF proteomic analyses align with molecular findings by showing an acute downregulation followed by later upregulation of myelin proteins such as Mbp and Plp1 after ketamine administration (Herzog et al., 2021). Noteworthy, behavioral studies support a connection between myelin remodeling and antidepressant efficacy (Weckmann et al., 2018; Pascual-Antón et al., 2021; Huang et al., 2023). Indeed, the sustained antidepressant effects of ketamine may depend on myelin-associated proteins like Mobp and involve AMPA receptor-mediated OPC differentiation (Huang et al., 2023).

Emerging evidence highlights the role of the gut-brain axis: while microbiome depletion alone did not impair remyelination in the cuprizone model, ketamine’s modulation of gut microbiota composition and increased lactic acid production correlated with enhanced remyelination and reduced Iba + immunofluorescence in the corpus callosum, suggesting attenuated microglial activity (Wang et al., 2022). These findings suggest a novel mechanistic link between ketamine, the microbiome, and myelin repair, opening promising avenues for microbiome-targeted adjunct therapies in neuropsychiatric treatment.

At higher or repeated doses, ketamine appears to exert detrimental effects on white matter integrity. Human neuroimaging studies have documented reductions in fractional anisotropy (FA) in frontal and temporoparietal white matter regions among chronic ketamine users, with changes correlating to cumulative dose (Liao et al., 2010; Edward Roberts et al., 2014). Additionally, decreases in axial diffusivity and disrupted connectivity within prefrontal circuits have been associated with dissociative symptoms in these populations (Edward Roberts et al., 2014). Structural white matter abnormalities also include increased WM volumes in individuals using ketamine alongside stimulants, indicating potentially complex interactions influenced by polydrug use (Liang et al., 2020). However, some investigations have reported no significant differences in WM measures compared to controls (Chesters et al., 2022), highlighting variability in findings.

These mixed results underscore the importance of considering dose, frequency, and comorbid substance use when evaluating ketamine’s impact on brain microstructure. The neurotoxic effects observed in chronic users (Liao et al., 2010; Wang et al., 2013; Edward Roberts et al., 2014; Liang et al., 2020; Chesters et al., 2022) contrast with ketamine’s pro-myelinating and neuroprotective actions at therapeutic doses (Vasavada et al., 2016; Sydnor et al., 2020; Taraku et al., 2023), suggesting a dose-dependent duality in ketamine’s influence on WM. This duality may reflect underlying mechanisms such as excitotoxicity or oxidative stress at higher exposures, potentially overriding beneficial signaling pathways involved in myelin maintenance. Future studies should prioritize longitudinal designs and detailed assessments of polysubstance interactions to clarify the threshold at which ketamine shifts from therapeutic to neurotoxic effects, informing safer clinical use and harm reduction strategies.

Animal and neonatal models further confirm WM vulnerability to early or repeated ketamine exposure. Adolescent and neonatal rodents show reduced myelin protein levels, compromised myelin sheath integrity, and altered OPC differentiation mediated by pathways such as PPARα and PI3K/Akt (Liu et al., 2024; Shan et al., 2025). High-dose neonatal S-ketamine causes sustained microstructural changes in corpus callosum with functional impairments (Zhou et al., 2025). Sex-specific alterations in myelin density and behavior were also reported following neonatal ketamine (Zhang et al., 2019). Future research should prioritize elucidating the developmental windows of vulnerability and molecular basis for the sex differences in ketamine’s effects on myelination.

Ketamine additionally modulates electrophysiological properties of myelinated axons, reducing sodium and potassium currents, which may contribute to functional disruptions (Arhem and Rydqvist, 1986). *In vitro*, ketamine induces apoptosis in OPCs and promotes pro-inflammatory astrocyte and microglia responses (Penning et al., 2021), while lower doses do not reduce OL numbers in neural stem cell cultures (Slikker et al., 2015).

Collectively, these findings highlight a complex, dose- and developmental stage-dependent effect of ketamine on myelin and white matter integrity. While low to moderate doses in adult or pathological contexts may foster myelin repair and plasticity, early-life or repeated high-dose exposure carries risks of lasting WM disruption, potentially mediated by neuroinflammation and altered OPC dynamics. The observed sex differences and involvement of specific signaling pathways suggest nuanced mechanisms underlying vulnerability, emphasizing the need for research assessing critical biological variables during key developmental windows. A thorough understanding of ketamine’s biphasic effects on myelin biology will be essential to maximize therapeutic benefits while mitigating potential neurotoxic outcomes.

### 4.2 Known effects of serotonergic RAADs on myelin

While ketamine has been the primary focus of research on RAAD-induced myelin plasticity, serotonergic RAADs—including LSD, psilocybin, and related tryptamines—also show growing therapeutic potential. These compounds predominantly act through the 5-HT system but may influence myelination via additional, less well-characterized mechanisms, such as 5-HT1A and 5-HT2A receptors on OPCs and OLs (Fan et al., 2015). This section synthesizes available data—ranging from biochemical studies of LSD–myelin interactions to cellular and imaging evidence—on how serotonergic psychedelics may modulate myelin integrity, structure, and function.

Mbp was reported to contain a 5-HT binding site in its tryptophan-rich domain, with both serotonin and LSD competing for this site *in vitro* and *in vivo* (Carnegie et al., 1972; Ishitani et al., 1978b). These interactions were hypothesized to occur at the nodes of Ranvier, potentially influencing membrane stability and immune recognition (Dickinson et al., 1970; Carnegie et al., 1972). Despite suggestive early evidence—such as recovery of LSD from myelin-rich brain fractions (Snyder and Reivich, 1966; Faragalla, 1972)—subsequent reports offered conflicting findings (Farrow and Van Vunakis, 1972, 1973). Methodological limitations in subcellular fractionation and the absence of modern molecular confirmation limit the interpretation of these data. Nonetheless, studies showing reduced leukocyte reactivity to MBP after exposure to indole psychedelics in individuals with neurodegenerative conditions (Carnegie et al., 1972) suggest potential immunomodulatory effects warranting further exploration.

Complementing this historical evidence, serotonergic RAADs may also influence myelination through their primary pharmacological target—the 5-HT system. OLs, OPCs, and Schwann cells express various 5-HT receptors (Yoder et al., 1997; Gaietta et al., 2003; Fan et al., 2015) and 5-HT immunoreactivity has been detected within myelinated axons in primate forebrain tracts (Azmitia and Gannon, 1983). Given the structural similarity between 5-HT and psychedelic tryptamines (Nichols, 2012), these compounds may mimic the actions of 5-HT or interfere with local serotonergic signaling in WM.

*In vitro* studies reveal that prolonged 5-HT exposure produces dose-dependent toxicity in OL lineage cells, reducing myelin protein expression and altering morphology (Fan et al., 2015). At high concentrations, 5-HT induced complete cell death in immature OLs. Similarly, DOI, a 5-HT2A receptor agonist, caused immature OLs and OPC cell death at higher doses—an outcome partially preventable by pre-treatment with the 5-HT2A antagonist ritanserin—indicating 5-HT2A receptor-mediated cytotoxicity (Fan et al., 2015). In co-culture systems, 5-HT impaired internode formation and disrupted contactin-associated protein (Caspr)-positive paranodal clustering, suggesting potential compromise to node of Ranvier organization (Fan et al., 2015).

However, *in vivo* findings present a more nuanced picture. Repeated administration of LSD increased cFos expression in Olig1 + OL lineage cells in the deeper layers of the rat PFC (Reissig et al., 2008), while also inducing methylation changes at the promoter of the zinc-finger transcription factor and transcriptional modulator yin yang 1 (YY1) (Inserra et al., 2022), which plays an important role in OPCs and OLs differentiation (He et al., 2007; He et al., 2010), and is a modulator of myelin-related gene expression in OLs and Schwann cells (Berndt et al., 2001; Zolova and Wight, 2011).

Yet, concerns remain regarding long-term and developmental exposure. Widespread microstructural changes were observed following repeated LSD administration during adolescence, including decreased FA in key WM tracts (Harris-Blum et al., 2024). Psilocybin-treated adult mice showed altered tract length and reduced neurite density in hippocampal and striatal WM regions (Frautschi et al., 2025). While these findings may point toward neuroplastic remodeling, further histological and ultrastructural validation, particularly by electron microscopy, would help clarify their precise implications for myelin organization.

Together, these findings suggest that serotonergic RAADs may modulate myelin integrity through several interrelated mechanisms: (a) direct binding to MBP and modulation of myelin protein–immune interactions; (b) serotonergic receptor-level modulation of OL and OPC activity; (c) transcriptional and activity-dependent changes that may promote adaptive myelination *in vivo*; and microstructural remodeling observed in neuroimaging studies, requiring histological validation to confirm myelin-specific effects. Whether these alterations constitute therapeutic neuroplasticity or maladaptive remodeling likely depends on dose, developmental timing, and biological context. Importantly, while *in vitro* findings emphasize dose-dependent cytotoxicity, *in vivo* outcomes appear more heterogeneous—likely shaped by complex interactions involving receptor desensitization, neuroimmune responses, and compensatory plasticity.

Given the evolving understanding of the 5-HT system (Moncrieff et al., 2023) and serotonergic RAADs (Ko et al., 2023) in depression, a re-evaluation of how RAADs modulate 5-HT signaling—and in turn, myelin integrity—is warranted. Longitudinal, dose-controlled studies using quantitative histology (e.g., MBP/PLP staining, EM) are critically needed to determine the net effects of serotonergic RAADs on WM plasticity and integrity across clinical and developmental/aging contexts.

### 4.3 Known and potential gut microbiome/metabolome-mediated effects of RAADs on myelin

Beyond their effects on the brain, RAADs may also influence myelin biology indirectly via the GM and GBA (Getachew et al., 2018; Wang et al., 2022; Inserra et al., 2023; Kelly et al., 2023; Campanale et al., 2024a; Reed and Foldi, 2024), a possibility that remains largely speculative. To date, only one published study (Wang et al., 2022) has directly examined RAAD-induced changes in the microbiome in relation to remyelination, highlighting the need for further research in this rapidly emerging field.

The GM and the GBA contribute to myelin homeostasis by regulating several pathways: (a) the production of pro-myelinating factors (Chen et al., 2019b; Keogh et al., 2021), (b) immunoendocrine, such as modulation of the hypothalamic–pituitary–adrenal (HPA) axis (Frankiensztajn et al., 2020), (c) vagal signaling (Tang et al., 2024), (d) neurometabolic pathways such as insulin-like growth factor 1 (IGF-1) signaling (Lu et al., 2018), and (e) gut-derived metabolites, including short-chain fatty acids (SCFAs) amino acids, indole derivatives, and lipid mediators (Lynch et al., 2021; Su et al., 2025).

Firstly, the GM contributes to myelin homeostasis through the production of metabolites that act as pro-myelinating factors along the gut–brain axis. Among these, the SCFA butyrate was demonstrated to play a central role in experimental demyelination models. Here it directly enhanced OL differentiation and promoted remyelination independently of microglial activity (Chen et al., 2019b). Early-life studies further confirmed this relationship: neonatal antibiotic showed to have long-term effects not only on the GBA but also on myelin modulation by increasing PFC myelin gene expression, consequently influencing cognitive outcomes (Keogh et al., 2021),

Secondly, the GM contributes to myelin homeostasis through immunoendocrine pathways, particularly by modulating the HPA axis. Early-life dysbiosis altered the normal development and functioning of the HPA axis, leading to changes in gene expression within key stress-regulatory regions such as the hippocampus and the paraventricular nucleus of the hypothalamus. Because HPA-axis activity governs systemic glucocorticoid release—hormones known to influence OL differentiation and myelin regulation—microbial disturbances that reshape HPA-axis responsiveness can indirectly affect myelin maintenance and plasticity axis (Buckley et al., 2020).

Also, Tang et al. (2024) proposed that the vagus nerve may mediate GM effects on OLs, potentially influencing myelin-related processes. This is supported by the study by Wang et al. (2023), who showed that in cuprizone-treated mice, transection of the subdiaphragmatic vagus nerve reduced demyelination, attenuated microglial activation, and partially restored GM composition, indicating that vagal signaling mediates key aspects of GM–driven effects on brain myelin and neuroinflammation.

Furthermore, the GM can influence myelin homeostasis by modulating neurometabolic pathways, particularly IGF-1 signaling. Studies in germ-free mice colonized with GM from preterm infants showed that a low-growth-associated microbiota reduced myelination, as indicated by lower myelin basic protein expression, and altered IGF-1 signaling, including decreased circulating and brain IGF-1 levels. These findings suggest that the GM can affect OL development and myelin formation through changes in IGF-1–mediated neurometabolic pathways (Lu et al., 2018).

Lastly, among the gut-derived metabolites, SCFAs such as acetate, propionate, and butyrate are the most robustly linked to central myelin regulation. Produced by bacterial fermentation of dietary fibers, these metabolites cross the blood–brain barrier (BBB) via monocarboxylate transporters (MCT1 and MCT4) expressed on brain endothelial cells (Uchida et al., 2011; Vijay and Morris, 2014). Once within the CNS, SCFAs influence myelination by modulating OL differentiation and maturation, promoting remyelination, and supporting a homeostatic microglial phenotype (Chen et al., 2019a; Calvo-Barreiro et al., 2021; Su et al., 2025). Lactic acid, derived from both host and brain metabolism and microbial fermentation, also supports OL maturation and myelin formation (Rinholm et al., 2011; Ichihara et al., 2017; Wu et al., 2023; Kim et al., 2025). Importantly, the relationship between the GM and myelin is bidirectional: not only do gut-derived metabolites such as SCFAs and lactic acid influence myelination, but myelin disruption itself has been shown to affect gut permeability, for example following bilateral hippocampal lysolecithin injection (Bostancıklıoğlu et al., 2023), indicating a dynamic interplay between myelination and peripheral microbial ecosystems.

In the only study directly connecting ketamine-induced microbiome changes to remyelination, ketamine enhanced remyelination in the cuprizone model, and this was associated with restoration of GM composition, increased lactic acid production, and reduced microglial activity (Wang et al., 2022). Interestingly, GM depletion alone did not impair remyelination, suggesting that ketamine’s pro-myelinating effects may be partially microbiota-dependent, though not exclusively mediated by gut signals. More broadly, preclinical studies show that RAADs like ketamine modulate the GM: in chronic pain models, repeated ketamine restored microbiota diversity, increased beneficial genera (e.g., *Bifidobacterium, Faecalibaculum, Romboutsia*), and reduced harmful taxa (*Pseudomonas, Serratia*), correlating with elevated fecal butyrate, normalized hippocampal BDNF, and improved cognitive function (Jiang et al., 2024).

Further studies show similar microbiome remodeling after ketamine administration in models of systemic inflammation (Huang et al., 2019) depression-like states (Getachew et al., 2018) and estrogen deficiency (Wan et al., 2022), with microbial shifts generally favoring anti-inflammatory and neuroprotective profiles. Given the close ties between inflammation, myelin degeneration, and psychiatric conditions, these ketamine-induced microbiota changes may secondarily impact myelination, though this remains mostly speculative. Data on serotonergic RAADs such as LSD remain scarce but suggest similar microbiome involvement. Repeated LSD increased *Bifidobacterium* and other beneficial taxa, altered tryptophan metabolism toward the serotonin pathway, and decreased kynurenine, a pathway implicated in OL dysfunction and myelin loss (Fathi et al., 2022; Inserra et al., 2023; Polyák et al., 2023; Campanale et al., 2024b; Hoseini and Ghafari, 2025).

In contrast, indole derivatives such as indole-3-propionate, indole-3-acetate, and indole-3-aldehyde—generated through bacterial tryptophan metabolism—are thought to access the brain via lipophilic diffusion or amino acid transporters, where they activate aryl hydrocarbon receptors (AHR) expressed on microglia and OLs. Activation of AHR modulates cellular signaling pathways involved in cell survival, differentiation, and inflammatory regulation, which in turn supports myelin repair and structural integrity (Rothhammer et al., 2016; Wang et al., 2023). Although these findings are mechanistically compelling, direct evidence linking RAADs to remyelination via microbiome-GBA interactions remains largely hypothetical.

### 4.4 Putative indirect effects of RAADs on myelin through neuronal activity–driven adaptive myelination and neurotrophic signaling

One possible mechanism underlying the effects of RAADs on myelin biology involves changes in neuronal electrical activity, an established driver of OPC proliferation and differentiation, and OL activity through bidirectional communication between active neurons and OPCs (Barres and Raff, 1993; Demerens et al., 1996; Gibson et al., 2014a; Kato and Wake, 2019). Hence, RAADs may not exert separate, independent effects on myelination, neuronal activity, neuroplasticity, and neurogenesis, but rather induce a broader, integrated state of global brain meta-plasticity. In this framework, enhancements in synaptic plasticity, neurogenesis, neuronal activity, and myelination are interconnected processes contributing together to the overall brain remodeling. By activating specific receptors on discrete neuronal populations in specific brain regions, RAADs may stimulate neuronal activity-dependent myelination. For example, selective activation of pyramidal neurons in corticolimbic circuits could enhance neuronal firing and synaptic activity, thus increasing the release of neurotransmitters and neurotrophic factors such as glutamate, ATP, and BDNF (De Gregorio et al., 2021; Inserra et al., 2021a; Inserra et al., 2021b; De Gregorio et al., 2022; Onofrj et al., 2023). These molecules activate specific receptors on OPCs and OLs, modulating their proliferation, differentiation, and the formation and remodeling of myelin sheaths (Stevens et al., 1998; Matsuda et al., 2009; Gibson et al., 2014b; de Faria et al., 2019; Fiore et al., 2023). Consequently, the changes in myelination observed with RAADs may not be direct or isolated effects but could result from the upstream induction of neuronal activity, neuroplasticity and neurogenesis (Catlow et al., 2013; Ly et al., 2018; Raval et al., 2021; Shao et al., 2021; Ornelas et al., 2022; Du et al., 2023; Moliner et al., 2023; Vargas et al., 2023). However, the precise cellular and molecular mechanisms through which RAADs modulate neuronal activity to influence OPC maturation, OL function, and activity-dependent myelination remain largely unresolved.

BDNF signaling through the target receptor TrKB in OPCs and OLs is essential for the regulation of appropriate myelin ensheathment and thickness during development. It also plays a crucial role in maintaining myelin plasticity and integrity in the adult brain (Wong et al., 2013; Fletcher et al., 2018). BDNF was shown to increase NMDA receptor currents in neurons by upregulating NR2C subunits, and to promote OL differentiation and Mbp expression via TrkB signaling (Suzuki et al., 2005; Vondran et al., 2010).

Recent evidence has revealed a direct pharmacological interaction between RAADs and TrkB signaling. Specifically, RAADs act as positive allosteric modulators of the TrkB receptor (Moliner et al., 2023), and increase the levels of endogenous BDNF (Ju et al., 2017; Wu et al., 2020). By simultaneously enhancing NMDA receptor activity, RAADs could potentiate TrkB-driven OL differentiation and myelin production, although in some contexts, excessive NMDA activation might antagonize TrkB-mediated effects depending on dose, developmental stage, or cell type. Future studies using combined TrkB and NMDA pathway manipulations are required to clarify these interactions and their contribution to myelin plasticity. Through this interplay between RAAD-mediated enhancement of TrkB signaling and NMDA receptor modulation, OL differentiation and myelin protein expression are likely promoted, contributing to adaptive myelin remodeling and WM plasticity in response to RAADs.

### 4.5 Putative indirect effects of RAADs on myelin through effects of microglia

Microglia are the innate resident macrophages of the brain and function as the primary immune defense against pathogens, injury, and disease. Beyond their immune functions, microglia play key roles in brain development, synaptic plasticity, homeostasis, and myelin dynamics, including formation and repair (Hagemeyer et al., 2017; Wlodarczyk et al., 2017; Hughes and Appel, 2020; Kent and Miron, 2024). Although microglia are not essential for the initial formation of myelin ensheathment by OLs, they are critical for the maintenance and regeneration of myelin and associated cognitive functions. Microglial influence on myelin biology occurs through multiple mechanisms, including: (a) phagocytosis of viable OPCs, preventing their differentiation into OLs (Kent and Miron, 2024), (b) activity-dependent engulfment of newly-formed myelin sheaths, fine-tuning myelination patterns in response to neuronal signaling (Hughes and Appel, 2020; Djannatian et al., 2023), (c) regulating OPCs migration to and proliferation/maturation into OLs at demyelination sites (Franklin and Ffrench-Constant, 2017), (d) modulating OL lipid metabolism via the transforming growth factor Beta 1 (TGFβ1)–TGFβ1 Receptor signaling axis (McNamara et al., 2023), (e) contributing to the clearance of myelin debris and participating in the phagocytosis of damaged myelin following injury or demyelination (Lloyd and Miron, 2019; Yong, 2022).

Given that RAADs activate anti-inflammatory and immunomodulatory signaling pathways (Loix et al., 2011; Nau et al., 2014; Szabo et al., 2014; Szabo et al., 2016; Flanagan and Nichols, 2018; Flanagan et al., 2019a; Flanagan et al., 2019b; Mason et al., 2023), it seems likely that RAADs could have a beneficial effect on myelin homeostasis and regeneration. This might be done indirectly by promoting transcriptional anti-inflammatory and immunomodulatory programs and their beneficial effects on microglia. Supporting this hypothesis, ketamine was shown to decrease Iba1 immunoreactivity in the corpus callosum and pathological scores in a mouse model of MS (i.e., experimental autoimmune encephalomyelitis) (Wang et al., 2021). Further evidence for microglial involvement comes from a study in which repeated ketamine treatment in mice exposed to cuprizone, resulted in decreased IBA1 immunoreactivity and increased myelination in the corpus callosum (Wang et al., 2022). Together, these findings support the notion that microglia play a fundamental role in maintaining and regenerating myelin, and that RAADs—through anti-inflammatory and immunoregulatory effects—may exert a protective influence on this microglia-mediated regulation of myelin integrity.

### 4.6 Putative effects of RAADs on myelin through the sigma-1 receptor

The sigma-1 receptor (Sig-1R), a multi-functional chaperone located at the mitochondria-associated ER membrane, regulates intracellular calcium homeostasis and trophic signaling and it is involved in myelin and neuronal plasticity and protection (Kourrich et al., 2012). The Sig-1R is a target receptor of DMT and 5-MeO-DMT (Fontanilla et al., 2009). It plays a significant role in myelination and myelin homeostasis through (a) promoting OPCs differentiation into mature OLs (Hayashi and Su, 2004a; Hayashi and Su, 2004b), (b) regulating the synthesis of myelin proteins and lipids necessary for the formation and maintenance of myelin (Hayashi and Su, 2004a; Ruscher and Wieloch, 2015), (c) modulating myelin structural and functional plasticity (Song et al., 2023), (d) eliciting protective effects, fostering OLs survival and myelin integrity (Lisak et al., 2020; Mukherjee et al., 2020; Song et al., 2023).

Although no direct evidence currently shows that RAADs or serotonergic psychedelics enhance myelination through Sig-1R activation, several studies have detailed mechanistic cascades by which Sig-1R influences OL and OPC physiology. Sig-1R, a chaperone located at the mitochondria-associated ER membrane, regulates intracellular calcium homeostasis and trophic signaling. Activation of Sig-1R initiates a molecular cascade that includes: (a) stabilization of IP3 receptors and increased ER–mitochondrial Ca^2+^ transfer, promoting mitochondrial ATP production necessary for myelin protein and lipid synthesis (Hayashi and Su, 2007); (b) modulation of Akt and ERK signaling (Wang et al., 2019), pathways that are well-established drivers of OPC differentiation and OL survival; (c) upregulation of neuroprotective and pro-plasticity transcriptional programs, partly via modulation of calcium-responsive transcription factors (e.g., CREB), which can influence expression of myelin-related genes (Ji et al., 2017); (d) attenuation of ER stress and unfolded-protein response signaling, thereby supporting OL survival under inflammatory or metabolic stress (Hayashi and Su, 2007; Mori et al., 2013); (e) regulation of cytoskeletal dynamics, potentially contributing to myelin sheath remodeling and functional plasticity (Hayashi and Su, 2001).

**Figure 2:**
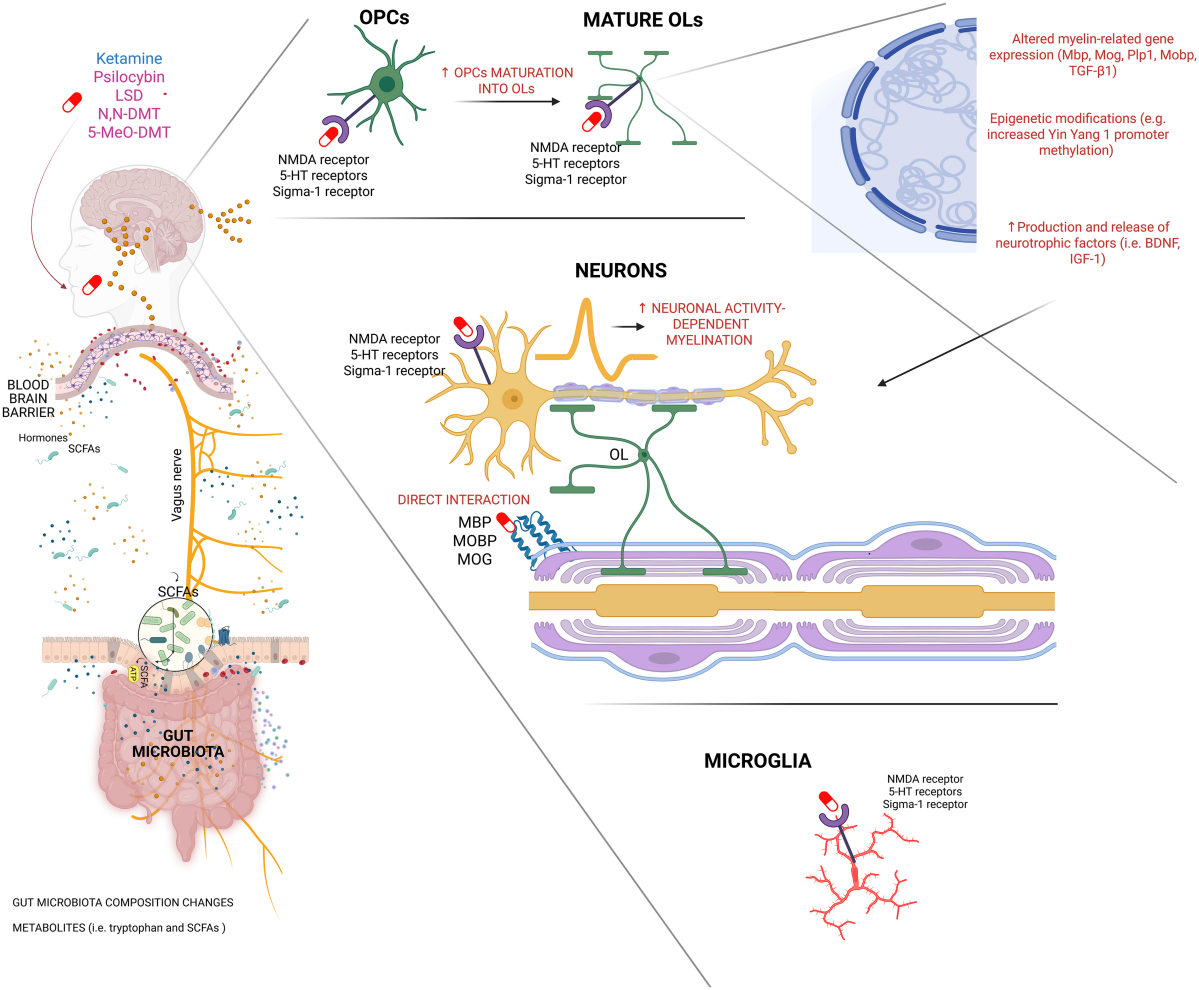
Proposed unifying conceptual model of how rapid-acting antidepressants (RAADs) may influence myelin plasticity. This figure illustrates a conceptual framework integrating several overlapping mechanisms through which RAADs—including ketamine, psilocybin, lysergic acid diethylamide (LSD), N, N-dimethyltryptamine (N, N-DMT), and 5-methoxy-N, N-dimethyltryptamine (5-MeO-DMT)—could modulate oligodendrocyte lineage cells, neuronal activity, and glial function to support adaptive myelination. RAADs act on receptors expressed across cell populations implicated in myelin plasticity, including N-methyl-D-aspartate (NMDA) receptors, serotonin (5-HT) receptors, and sigma-1 receptors on oligodendrocyte precursor cells (OPCs), mature oligodendrocytes (OLs), neurons, and microglia. Increased neuronal activity following RAAD administration may promote activity-dependent myelination, while receptor-mediated signaling can facilitate OPC maturation into OLs. RAADs may also influence myelin homeostasis through modulation of myelin-related gene expression—including Mbp (myelin basic protein), Mog (myelin oligodendrocyte glycoprotein), Plp1 (proteolipid protein 1), Mobp (myelin-associated oligodendrocyte basic protein), and Tgfb1 (transforming growth factor beta 1)—as well as through epigenetic mechanisms such as altered promoter methylation (e.g., Yin Yang 1). Additional pathways include increased production and release of neurotrophic factors such as brain-derived neurotrophic factor (BDNF) and insulin-like growth factor-1 (IGF-1), which may enhance glial plasticity. Finally, the model incorporates gut–brain axis interactions, including RAAD-related shifts in gut microbiota composition, short-chain fatty acid (SCFA) and tryptophan metabolite signaling, and vagal pathways that may modulate microglial tone and myelin dynamics. Together, these domains represent a speculative but biologically plausible, multi-level framework through which RAAD exposure could influence myelin plasticity in a context- and activity-dependent manner. Created with BioRender.

As such, it could be speculated that the Sig-1R agonists DMT and 5-MeO-DMT might affect OPCs and OLs through Sig-1R receptor signaling, putatively modulating myelin homeostasis, in addition to their known anti-inflammatory (Szabo et al., 2014; Szabo et al., 2016) and neurogenesis-enhancing effects (Morales-Garcia et al., 2020). To date however, no direct evidence exists that DMT and 5-MeO-DMT affect myelination via Sig-1R receptor signaling (see Figure 2).

### 4.7 Future directions

While preliminary evidence suggests that specific regimens of RAADs may positively influence myelination and OL function, these effects are context-dependent and may not be universally beneficial. Activity-dependent myelination could strengthen neural circuits that underlie maladaptive behaviors or pathophysiology, potentially reinforcing undesirable connectivity. It is also plausible that some RAADs might impair aspects of myelination under certain conditions, such as overuse, or disrupt the finely tuned balance of myelin plasticity, particularly with chronic or repeated exposure, such as in long-term microdosing, a largely unexplored area.

Moreover, the long-term consequences of modulating myelination, especially with chronic or repeated RAAD exposure (e.g., long-term microdosing), remain unclear and warrant caution. Vulnerable populations—including older adults, individuals with pre-existing myelin or neurodegenerative disorders, and those with comorbidities or altered immune function—may respond differently to RAADs and be at higher risk for adverse effects. Understanding safety and efficacy in these groups is critical before broader clinical application should be considered. Hence, clinical translation must proceed cautiously, with rigorous evaluation of risks and benefits.

Overall, the promising yet preliminary evidence of RAADs’ effects on myelin biology underscores the urgency and importance of investigating in future studies their safety and therapeutic potential in individuals with myelin- and neurodegenerative disorders. Myelin plasticity is highly context-dependent, and not all changes may be beneficial. The long-term consequences of modulating myelination—especially under conditions of chronic or repeated RAAD exposure—, especially in vulnerable populations such as aging individuals with neurodegenerative disorders remain unclear and warrant rigorous investigation.

In addition, sex, gender, and age are likely to influence both the baseline trajectory of myelination and its response to RAADs. Hormonal differences, particularly during puberty, reproductive years, and menopause/andropause, may modulate how RAADs are metabolized, and how OLs respond to pharmacological stimuli. Moreover, developmental stage and aging introduce distinct windows of vulnerability and plasticity, emphasizing the need for stratified analyses in both preclinical and clinical studies. Particular attention should be given to vulnerable populations, including older adults, individuals with existing myelin or neurodegenerative disorders, and those with comorbidities or altered immune function, as they may respond differently to RAAD treatment and be at higher risk for adverse events. Understanding the safety and efficacy profiles in these groups remains a critical step forward.

Future studies should also prioritize the GBA contributions to RAADs-induced myelin plasticity. This includes identifying specific microbiota taxa and metabolite changes—such as SCFAs, tryptophan metabolites, and KP intermediates—that mediate myelin integrity. Experimental approaches could involve fecal microbiota transplantation, germ-free models, pre- and probiotics administration, and metabolomics. Such work may uncover gut-derived biomarkers predictive of RAAD efficacy or clinical risk, paving the way for combined microbiome–RAADs precision therapeutic strategies for myelin homeostasis.

Future -*omics* and cell type-specific studies are essential to better understand how RAADs regulate genes and proteins involved in myelin synthesis, compaction, and maintenance, and how these changes evolve over time or following repeated use. Targeted studies using *in vivo* calcium imaging, cell type–specific manipulations, and time-resolved and cell-type specific transcriptomic or proteomic profiling will be instrumental in disentangling these pathways and determining the extent to which RAADs-induced myelination is causally linked to their therapeutic effects, while also accounting for contextual factors such as sex, age, and clinical background, which may modulate these mechanisms.

Microscopy and ultrastructural imaging could help clarify the precise myelin alterations (e.g., compactness, thickness, internode length) induced by different doses and regimens of RAADs over time, while non-invasive imaging methods such as DTI, tractography, myelin water imaging (MWI), and magnetization transfer imaging (MTI) could provide valuable complementary insights into WM microstructure and myelin content *in vivo*. Integrating longitudinal imaging, behavioral assessments, and multi-*omics* translational approaches across preclinical and clinical settings will be crucial to clarify whether these compounds promote adaptive or maladaptive forms of myelin remodeling. Crucially, the potential long-term consequences of modulating myelin dynamics—positive or negative—must be systematically evaluated.

## 5 Conclusion

This scoping review highlights preliminary evidence that RAADs, including ketamine and serotonergic psychedelics, can influence myelin biology through diverse dose-, region-, and context-dependent mechanisms. These include direct effects on OL lineage cells, modulation of serotonergic, glutamatergic, and neurotrophic signaling, neuronal activity-dependent myelination, gene expression changes, putative interaction with structural myelin proteins, and indirect pathways via the gut–brain axis. Exposure of humans and animals to therapeutic doses and regimens appears to promote myelin plasticity or repair, while excessive or juvenile exposures appears to lead to myelin disruption. Dose-finding and safety studies are warranted to clarify how RAAD influence myelin integrity and overall brain health, particularly across disorders, age groups, gender, and vulnerable populations. Systematic examinations using modern molecular and imaging techniques may uncover novel therapeutic targets for modulating myelin integrity. Randomized controlled trials are encouraged to define the boundaries of safety and efficacy. Overall, the therapeutic potential of RAADs represents a potentially promising, yet underexplored frontier in myelin health, with potential implications for neurology, psychiatry, and healthy aging.
